# Heme oxygenase 1 protects human colonocytes against ROS formation, oxidative DNA damage and cytotoxicity induced by heme iron, but not inorganic iron

**DOI:** 10.1038/s41419-020-02950-8

**Published:** 2020-09-23

**Authors:** Nina Seiwert, Sabine Wecklein, Philipp Demuth, Solveig Hasselwander, Talke A. Kemper, Tanja Schwerdtle, Thomas Brunner, Jörg Fahrer

**Affiliations:** 1 Department of Toxicology, University Medical Center, Mainz, Germany; 2 Rudolf Buchheim Institute of Pharmacology, Justus Liebig University Giessen, Giessen, Germany; 3 Division of Food Chemistry and Toxicology, Department of Chemistry, Technical University of Kaiserslautern, Kaiserslautern, Germany; 4 Department of Food Chemistry, Institute of Nutritional Science, University of Potsdam, Nuthetal, Germany; 5 TraceAge – DFG Research Unit on Interactions of Essential Trace Elements in Healthy and Diseased Elderly (FOR 2558), Berlin-Potsdam-Jena, Germany; 6 Biochemical Pharmacology, Department of Biology, University of Konstanz, Konstanz, Germany; 7 Present Address: Department of Pharmacology, University Medical Center, Mainz, Germany

**Keywords:** Colorectal cancer, Iron, DNA damage response, Oncogenesis

## Abstract

The consumption of red meat is probably carcinogenic to humans and is associated with an increased risk to develop colorectal cancer (CRC). Red meat contains high amounts of heme iron, which is thought to play a causal role in tumor formation. In this study, we investigated the genotoxic and cytotoxic effects of heme iron (i.e., hemin) versus inorganic iron in human colonic epithelial cells (HCEC), human CRC cell lines and murine intestinal organoids. Hemin catalyzed the formation of reactive oxygen species (ROS) and induced oxidative DNA damage as well as DNA strand breaks in both HCEC and CRC cells. In contrast, inorganic iron hardly affected ROS levels and only slightly increased DNA damage. Hemin, but not inorganic iron, caused cell death and reduced cell viability. This occurred preferentially in non-malignant HCEC, which was corroborated in intestinal organoids. Both hemin and inorganic iron were taken up into HCEC and CRC cells, however with differential kinetics and efficiency. Hemin caused stabilization and nuclear translocation of Nrf2, which induced heme oxygenase-1 (HO-1) and ferritin heavy chain (FtH). This was not observed after inorganic iron treatment. Chemical inhibition or genetic knockdown of HO-1 potentiated hemin-triggered ROS generation and oxidative DNA damage preferentially in HCEC. Furthermore, HO-1 abrogation strongly augmented the cytotoxic effects of hemin in HCEC, revealing its pivotal function in colonocytes and highlighting the toxicity of free intracellular heme iron. Taken together, this study demonstrated that hemin, but not inorganic iron, induces ROS and DNA damage, resulting in a preferential cytotoxicity in non-malignant intestinal epithelial cells. Importantly, HO-1 conferred protection against the detrimental effects of hemin.

## Introduction

Heme is an important iron source in human nutrition and occurs mainly in food of animal origin such as meat^1^. Heme b is the most abundant heme, consisting of a protoporphyrin ring with a central iron (Fe^2+^). As such, it is the prosthetic group of the oxygen transporter proteins hemoglobin and myoglobin^2^. The central iron is coordinated by four nitrogen atoms of the protoporphyrin backbone and a histidine residue of the globin, leaving one free ligand-binding site for oxygen^2^. Upon its dietary uptake, heme is liberated from myoglobin or hemoglobin due to the acidic pH in the stomach and the enzymatic proteolysis occurring in both the stomach and duodenum^3^. Heme is then internalized into intestinal epithelial cells, which is likely mediated by a transporter called heme carrier protein 1 (HCP-1; also known as proton-coupled folate transporter or PCTF)^4,5^. In the cytosol, it is finally catabolized by the enzyme heme oxygenase-1 (HO-1) to biliverdin, ferrous iron (Fe^2+^) and carbon monoxide (CO)^6^.

In recent years, the dietary uptake of heme iron has attracted great attention due to its involvement in the etiology of colorectal cancer (CRC)^7^. Heme occurs abundantly in red meat^8^, whose consumption has been classified as possibly carcinogenic to humans by the International Agency for Research on Cancer^9^. Several lines of evidence indicate that heme iron is the critical constituent of red meat driving colorectal tumorigenesis. The underlying mechanisms are not fully understood, but include intestinal hyperproliferation^10,11^, changes in the gut microbiota^12,13^ and genotoxic effects triggered by heme iron^14,15^. Apart from that, it has been shown that dietary heme promotes lipid peroxidation and, thus, the formation of reactive aldehydes such as 4-hydroxynonenal (4-HNE)^16,17^. Interestingly, fecal water obtained from heme-fed rodents or its constituent 4-HNE exhibited a higher toxicity in normal murine colon epithelial cells with wildtype APC as compared to pre-neoplastic cells bearing a mutated APC allele (APC^Min/+^)^17,18^. The cellular resistance to heme triggered cytotoxicity was mediated by key enzymes responsible for 4-HNE detoxification^19^ and by the activation of the transcription factor Nrf2^20^, which is known to be engaged by oxidative stress and electrophilic compounds^21^.

Surprisingly, the effects induced by heme iron have never been directly compared to that of inorganic iron in normal or transformed colonocytes, and it is still unclear whether heme iron or its cellular breakdown product ferrous iron is responsible for DNA damage and cytotoxicity. In this work, we studied the genotoxic and cytotoxic potential of heme iron versus inorganic iron in normal human colonic epithelial cells (HCEC), human CRC cells lines and murine intestinal organoids. Initially, the formation of reactive oxygen species (ROS) and induction of DNA damage was assessed. Next, the impact of both iron species on cell cycle progression, cell death induction and cell viability was monitored. Iron uptake in HCEC and CRC cells was measured by inductively coupled plasma mass spectrometry (ICP-MS) and, indirectly, via detection of HO-1 expression levels. Finally, we addressed the role of HO-1 in heme-triggered ROS formation, DNA damage and cytotoxicity in both HCEC and CRC cells using the HO-1 inhibitor zinc protoporphyrin (ZnPP) and siRNA mediated knockdown of HO-1.

## Results

### Heme iron, but not inorganic iron, promotes ROS production and oxidative DNA damage in HCEC and CRC cells

**Fig. 1:**
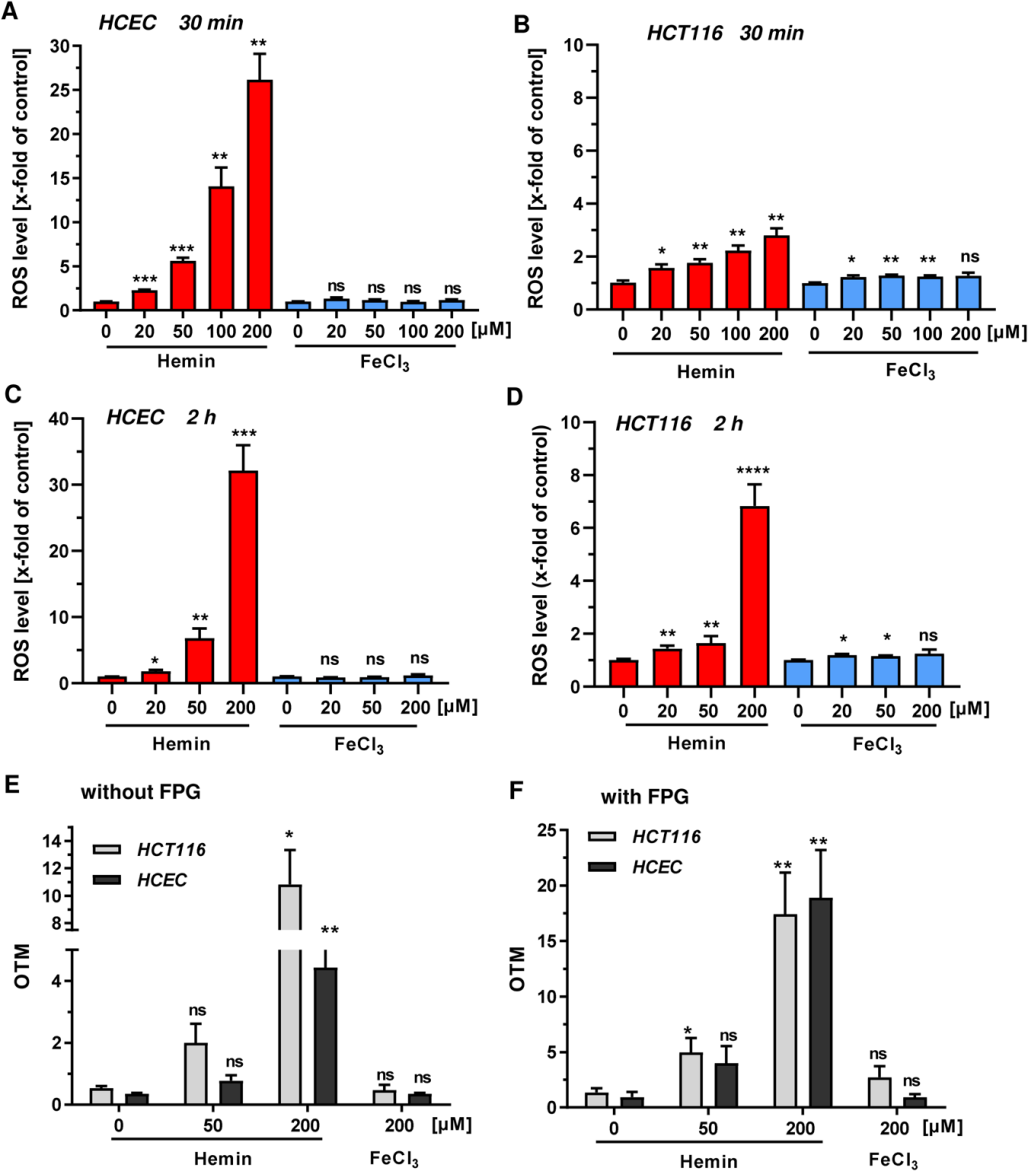
Time-dependent formation of ROS and oxidative DNA damage in HCEC and CRC cells by heme iron versus inorganic iron. **a**, **c** HCEC were incubated for 30 min (**a**) or 2 h (**c**) with increasing doses of hemin or FeCl_3_ (0–200 µM). Reactive oxygen species (ROS) levels were assessed by live cell staining and subsequent flow cytometry-based analysis. **b**, **d** HCT116 were treated for 30 min (**b**) or 2 h (**d**) and analyzed as described under **a**. Data (**a**–**d**) is shown as mean + SEM (*n* ≥ 3, except for 20 µM in HCT116 at 2 h: *n* = 2). Ns: *p* > 0.05; *p < 0.05; **p < 0.01; ***p < 0.001, ****p < 0.0001 (versus respective control). **e**, **f** Cells were exposed to hemin (0–200 µM) or FeCl_3_ (200 µM) for 2 h. DNA strand break induction and formation of oxidative DNA damage was determined by the alkaline Comet assay without (**e**) or with Fpg (**f**). Data are presented as mean + SEM (*n* ≥ 3). Ns: *p* > 0.05; *p < 0.05; **p < 0.01 (versus control).

First, we investigated the production of ROS in HCEC and CRC cell lines in response to hemin (Fe^3+^ protoporphyrin chloride) and ferric chloride (FeCl_3_) treatment using flow cytometry (Fig. 1a–d). In HCEC, hemin caused a dose-dependent increase in ROS levels already 30 min after incubation, with 200 µM hemin resulting in a 25-fold induction as compared to control cells (Fig. 1a and Supplementary Fig. S1A). The dose-dependent ROS production by hemin further increased after 2 h and was still observed after 24 h, albeit at reduced levels (Fig. 1c; Supplementary Figs. S1A and S2B). In contrast, ferric iron only slightly induced ROS formation independent of the incubation period (Fig. 1a, c and Supplementary Fig. S2B). Importantly, incubation of HCEC with ferrous iron (FeSO_4_) had also no or little effect on cellular ROS levels (Supplementary Fig. S2A, B). In HCT116 CRC cells, hemin caused an up to 3-fold upregulation of ROS levels as compared to control cells after 30 min (Fig. 1b and Supplementary Fig. S1B), with generally weaker effects as seen in HCEC. ROS levels further increased over time at the highest hemin dose (Fig. 1d, Supplementary Figs. S1B and S2D). Interestingly, FeCl_3_ hardly affected basal ROS levels in HCT116 cells (Fig. 1b, d and Supplementary Fig. S2D). A comparable hemin-dependent increase in ROS generation was detected in Caco-2 CRC cells, in which FeCl_3_ led to a non-significant rise in ROS levels (Supplementary Fig. S1C). Furthermore, ROS formation was determined in HCT116 cells maintained under reduced oxygen levels similar to HCEC. As observed before, hemin increased ROS production in HCT116 cells in a dose-dependent manner with slightly weaker effects as observed under standard culture conditions (Supplementary Fig. S2E).

We then set out to analyze the impact of hemin or FeCl_3_ on DNA damage induction using the alkaline Comet assay with or without the DNA glycosylase Fpg. This enzyme recognizes 8-Oxoguanine (8-oxoG) and catalyzes the excision of the damaged base via ß-δ elimination, resulting in the generation of DNA single-strand breaks^22^. Hence, this setup allows for the detection of both DNA strand breaks and oxidative DNA damage (i.e., 8-oxoG). Hemin significantly induced DNA strand breaks after 2 h of incubation in both HCT116 and HCEC (Fig. 1e). Interestingly, the Fpg-modified alkaline Comet assay revealed a strong dose-dependent induction of oxidative DNA damage in both cell types (Fig. 1f). In turn, inorganic iron generated neither substantial levels of oxidative DNA lesions nor DNA strand breaks (Fig. Fig. 1e, f), which is in line with the lack of ROS production (Fig. 1). After 24 h of incubation, DNA strand break level returned to baseline levels except for HCEC treated with 100 µM hemin (Supplementary Fig. S3A). Oxidative DNA damage was still observed after hemin treatment in HCT116 cells, although the levels were diminished as compared to those after 2 h (Supplementary Fig. S3B). In HCEC, a massive induction of oxidative DNA lesions was found at a dose of 100 µM hemin. It is noteworthy that FeCl_3_ showed almost no genotoxicity also after 24 h in both HCT116 cells and HCEC (Supplementary Fig. S3A, B). Furthermore, ferrous iron (FeSO_4_) was not genotoxic in HCEC (Supplementary Fig. S3C). Taken together, our results demonstrate that hemin, in contrast to inorganic iron, generates ROS, DNA strand breaks and oxidative DNA damage in both normal HCEC and CRC cells.

**Fig. 2:**
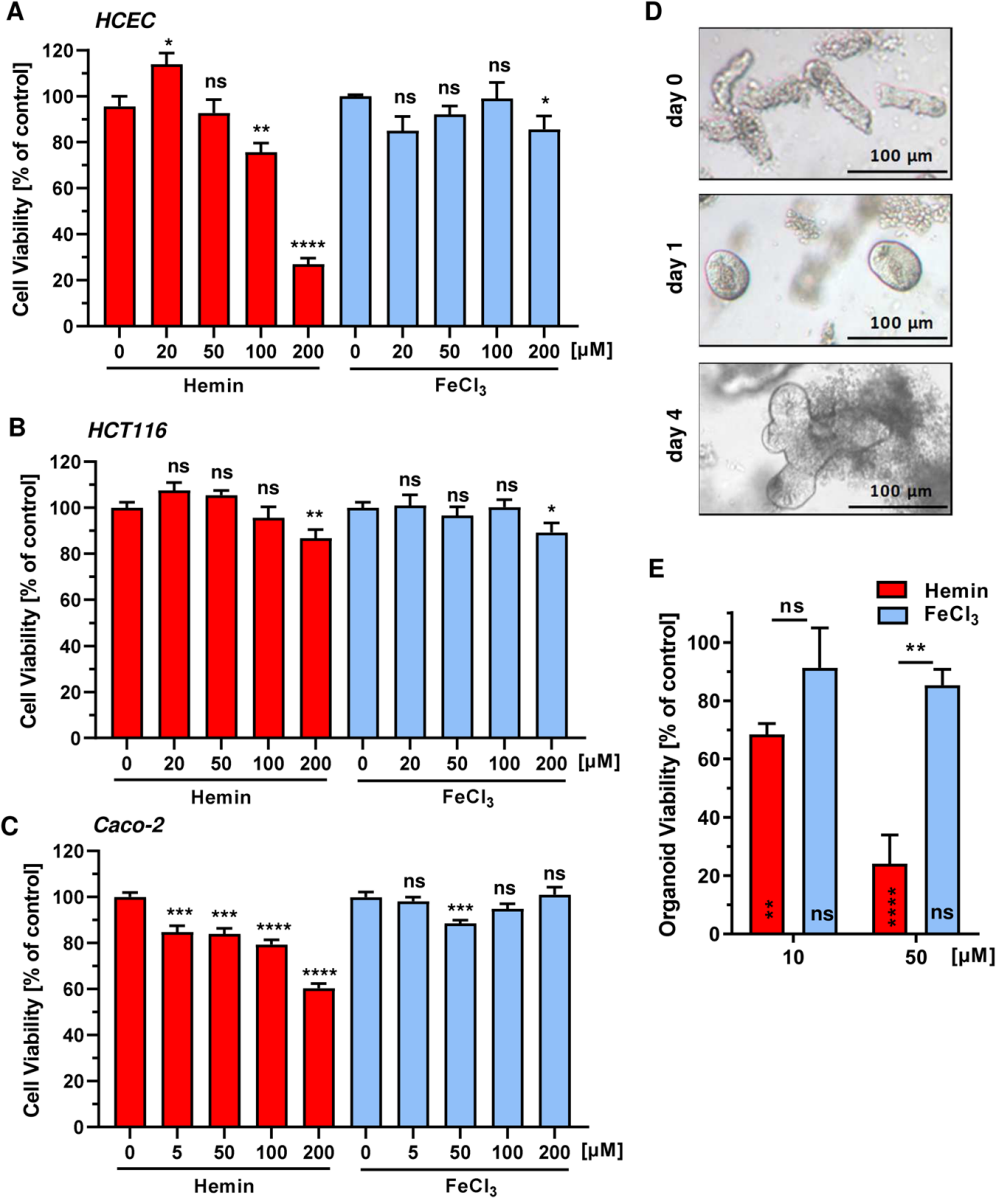
Impact of heme iron and inorganic iron on cell and intestinal organoid viability. **a**–**c** HCEC (**a**), HCT116 (**b**) and Caco-2 (**c**) were treated with increasing concentrations of hemin or FeCl_3_ (0–200 µM). Cell viability was determined after 72 h using the MTS assay. Data are given as mean + SEM (*n* ≥ 3, triplicates). Ns: *p* > 0.05; *p < 0.05; **p < 0.01; ***p < 0.001; ****p < 0.0001 (versus respective control). **d** Microscopic images of isolated intestinal crypts (day 0) and the developing intestinal organoids (day 1 and 4). **e** Intestinal organoids were treated with hemin or FeCl_3_ for 24 h and viability was determined by the MTT assay_._ Date are given as mean + SEM (*n* = 2). Ns: *p* > 0.05; **p < 0.01; ****p < 0.0001.

### Heme iron, not inorganic iron, impairs viability predominantly in HCEC and intestinal organoids

Having shown that hemin is genotoxic, its effects on cell viability were determined after 72 h using the MTS assay. Hemin led to a pronounced, dose-dependent reduction of viability in HCEC, reaching about 25% viability at 200 µM (Fig. 2a). HCT116 cells were generally more resistant towards hemin treatment and showed only a moderate decrease in viability at the highest hemin dose (Fig. 2b). Caco-2 CRC cells also exhibited a lower sensitivity towards hemin than HCEC, with a viability of 60% at 200 µM hemin (Fig. 2c). Interestingly, viability of HCEC and the CRC cell lines were only slightly reduced by inorganic iron (Fig. 2a–c and Supplementary Fig. S4E), consistent with its weak effects on ROS and DNA damage formation observed before. It should be noted that HCT116 and Caco-2 cells maintained under reduced oxygen tension (7% O_2_ like HCEC) displayed similar sensitivity towards hemin as compared to the standard culture conditions (Supplementary Fig. S4C, D). Further experiments revealed that other CRC cell lines (RKO, LS174T) were also more resistant towards hemin than HCEC (Supplementary Fig. S4A, B). Taken together, heme triggered cytotoxicity was higher in HCEC as compared to the different CRC cell lines tested, while FeCl_3_ hardly affected viability in all cell lines. Finally, murine intestinal organoids were established to study the effects of hemin versus FeCl_3_ on their growth and viability (Fig. 2d). Hemin caused a substantial reduction in organoid viability already at 10 µM, with a further potentiation at 50 µM (Fig. 2e). In contrast, FeCl_3_ only moderately decreased organoid viability, which closely mirrors the effects observed in HCEC (Fig. 2a, e). In conclusion, these findings provided evidence that hemin reduces viability predominantly in HCEC and intestinal organoids, while various CRC cell lines are only modestly affected. Moreover, inorganic iron displayed weak toxicity in all cell models and the intestinal organoids.

**Fig. 3:**
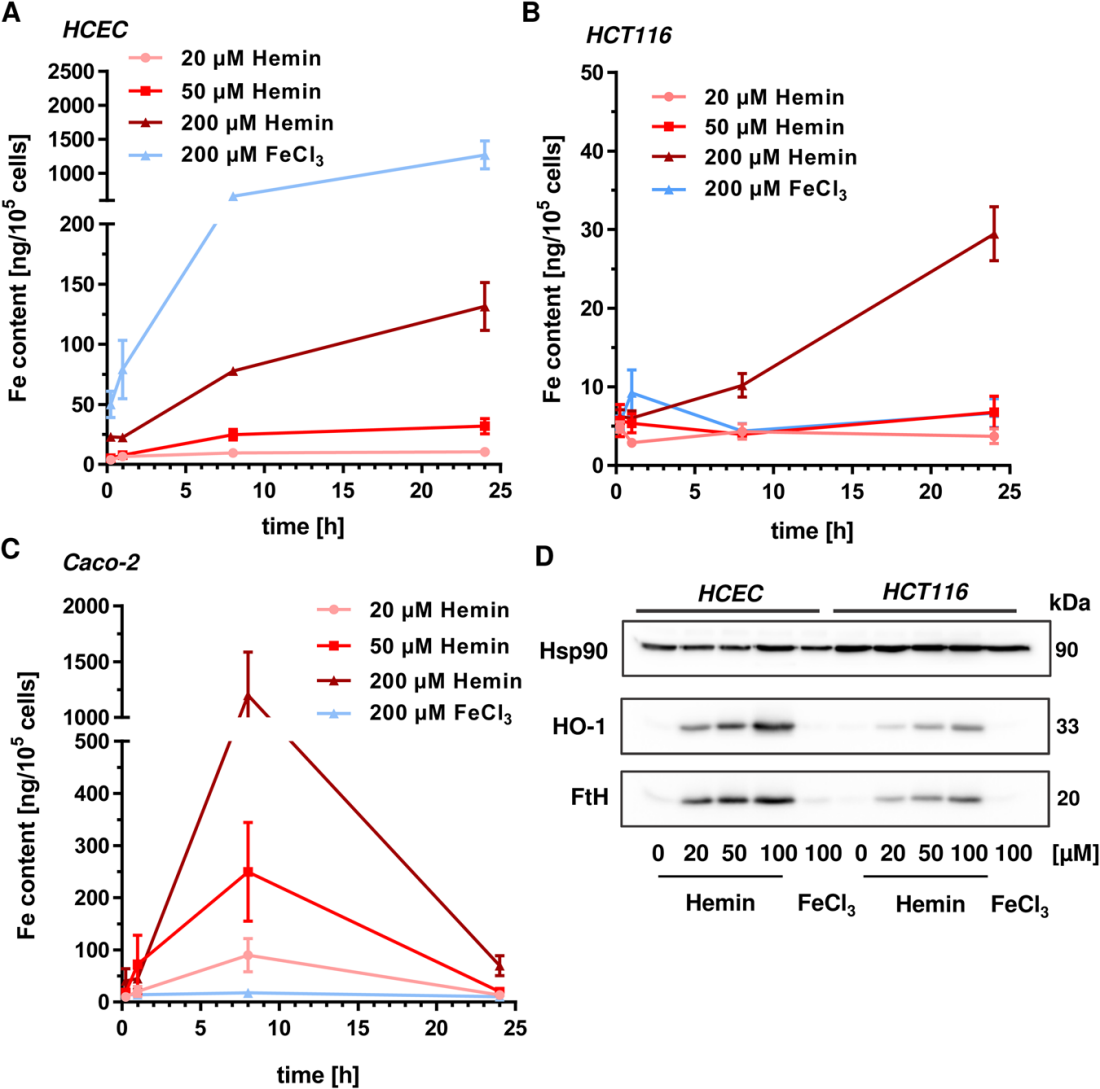
Uptake of hemin and inorganic iron into HCEC and CRC cells. **a** HCEC were incubated with hemin or FeCl_3_ for different periods (0, 0.25, 8 and 24 h) at the concentrations indicated. Cells were then collected and iron content was determined by ICP-MS as described. Data are depicted as mean + SEM (*n* ≥ 3). **b** HCT116 cells were treated with hemin or FeCl_3_ and iron content was analyzed as mentioned above. Data are shown as mean + SEM (*n* = 7). **c** Caco-2 cells were treated and analyzed as described under **a**. Data are presented as mean + SEM (*n* ≥ 6). **d** HCEC and HCT116 cells were exposed to hemin or FeCl_3_ for 24 h. Cells were harvested, lysed and subjected to Western blot analysis for heme oxygenase-1 (HO-1) and ferritin heavy chain gene (FtH). Hsp90 was detected as loading control.

### Uptake kinetics of heme iron and inorganic iron into HCEC and CRC cells

Next, we wished to know whether the cellular iron uptake might explain the observed differences between heme iron and inorganic iron on the one hand as well as HCEC and CRC cells on the other hand. To this end, cells were incubated with different hemin and FeCl_3_ concentrations for up to 24 h. Cells were then harvested and analyzed for their total cellular iron content (both free iron and iron bound to proteins) using ICP-MS. First, the iron content was assessed in HCEC, revealing a time- and dose-dependent increase of the iron content following hemin treatment (Fig. 3a). After 24 h, 200 µM hemin resulted in an iron content of 131 ng/10^5^ cells. Inorganic iron was internalized even more efficiently, with a maximum of 1200 ng iron/10^5^ cells after 24 h (Fig. 3a). In HCT116 cells, a time- and dose-dependent increase in cellular iron was detected upon hemin exposure, reaching a maximum Fe content (~30 ng/10^5^ cells) after treatment with 200 µM hemin for 24 h (Fig. 3b). Incubation with 200 µM FeCl_3_ resulted in a maximum Fe content already after 1 h, which then leveled out to about 7 ng iron/10^5^ cells after 24 h. As observed in HCT116 and HCEC, hemin treatment resulted in a dose-dependent increase of the cellular iron level in Caco-2 cells, with a maximum of 1200 ng Fe/10^5^ cells after 8 h (Fig. 3c). At the same time, incubation with 200 µM FeCl_3_ raised cellular iron levels to 17 ng/10^5^ cells. As another readout for hemin uptake, the protein expression of HO-1 was analyzed in cell lysates form HCEC and HCT116 after 8 and 24 h (Fig. 3d and Supplementary Fig. S5C). HO-1 is responsible for the intracellular breakdown of hemin to ferrous iron, biliverdin and CO^23^. No or very little HO-1 induction was detected after FeCl_3_ treatment in both cell types. In opposition to that, hemin upregulated HO-1 expression in a dose-dependent manner, with stronger effects in HCEC than in HCT116 cells (Fig. 3d and Supplementary Fig. S5C). This is likely attributable to the better internalization of hemin into HCEC, resulting in up to 6-fold higher iron content as compared to HCT116 (Fig. 3a vs. 3b). Furthermore, a dose-dependent upregulation of ferritin heavy chain (FtH) was observed after hemin exposure in both cell types, which was also more prominent in HCEC than in HCT116 cells and not induced by inorganic iron (Supplementary Fig. S5D, E). FtH and ferritin light chain (FtL) subunits assemble into the 24-mer ferritin (FTN), which is known to sequester free iron released by HO-1 mediated breakdown of heme^24^. In summary, heme iron and inorganic iron were taken up in a rather cell type-dependent manner. Furthermore, the cellular iron contents seem not to directly correlate with the observed genotoxic and cytotoxic effects.

**Fig. 4:**
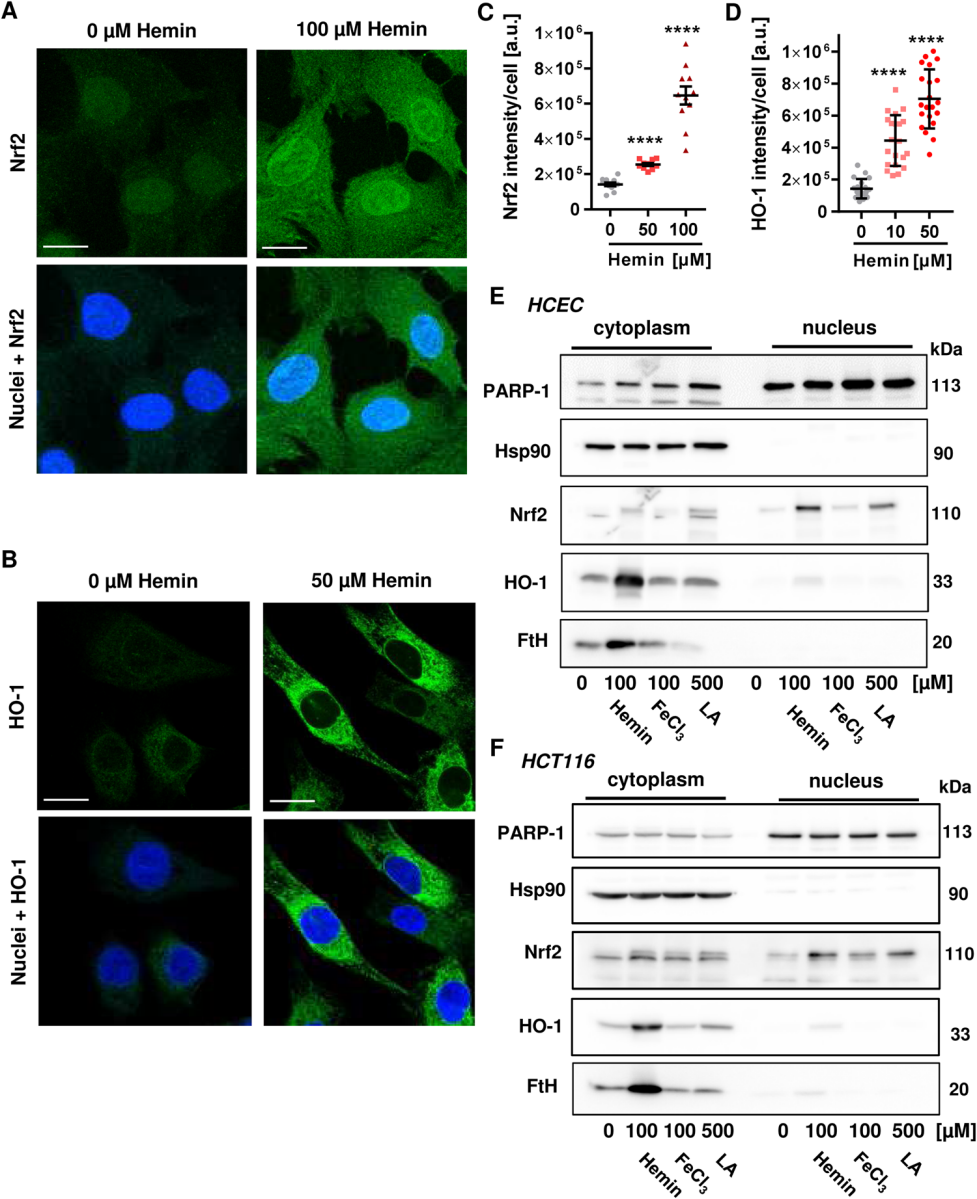
Impact of hemin and inorganic iron on Nrf2 signaling in HCEC and CRC cells. **a** HCEC were exposed to hemin (0–100 µM) for 2 h. Cells were fixed, processed for Nrf2 staining and analyzed by confocal microscopy. Representative images are shown. Nrf2 is depicted in green and nuclei are shown in blue. Scale bar: 20 µm. **b** HCEC were treated with hemin (0–50 µM) for 8 h. Cells were fixed, processed for HO-1 staining and analyzed by confocal microscopy. Representative images are shown. HO-1 is depicted in green and nuclei are shown in blue. Scale bar: 20 µm. **c** Quantitative evaluation of Nrf2 staining shown in **a**. Nrf2 intensity was quantified by ImageJ. Data are given as mean + SEM (*n* = 2). ****p < 0.0001. **d** Quantitative evaluation of HO-1 staining shown in **b**. HO-1 intensity was quantified by ImageJ and data are indicated as mean + SEM (*n* = 3). ****p < 0.0001. **e** HCEC were incubated for 8 h with 100 µM hemin, 100 µM FeCl_3_, or 500 µM α-Lipoic acid (LA). Cell fractionation was performed as described. Cytoplasmic and nuclear protein extracts were then analyzed by SDS-PAGE and western blot detection of Nrf2, HO-1 and FtH. PARP-1 served as nuclear loading control, while Hsp90 was used as cytoplasmic loading control. **f** HCT116 were treated, processed and analyzed as stated under **e**.

### Hemin activates Nrf2 signaling and stimulates HO-1 expression in HCEC and HCT116 cells

We then analyzed the activation of the transcription factor Nrf2 by hemin. Nrf2 is a major player in the cellular response to oxidative stress, which results in its stabilization and translocation from the cytosol into the nucleus^25^. Treatment of HCEC with increasing concentrations of hemin (0–100 µM) for 2 h led to increased Nrf2 staining intensity and its accumulation in the nucleus as visualized by confocal microscopy (Fig. 4a, c and Supplementary Fig. S5A). Furthermore, HO-1 expression and its subcellular localization were studied, revealing a dose-dependent increase in HO-1 intensity already detectable at 10 µM hemin after 8 h. As expected, HO-1 was detected almost exclusively in the cytoplasm of HCEC (Fig. 4b, d and Supplementary Fig. S5B). Moreover, the subcellular localization of Nrf2 and HO-1 was studied by Western blot analysis following cell fractionation. Consistent with confocal microscopy, hemin increased both Nrf2 and HO-1 levels in HCEC and promoted the accumulation of Nrf2 in the nuclear fraction. Likewise, hemin provoked FtH upregulation in the cytoplasm. In contrast, inorganic iron provoked modest Nrf2 induction without significantly upregulating HO-1 or FtH (Fig. 4e). LA was included as a known activator of Nrf2^26^, and thus caused Nrf2 stabilization with concomitant nuclear translocation. The lack of HO-1 induction by LA despite the observed Nrf2 activation is consistent with the notion that HO-1 induction by Nrf2 requires the inactivation of the transcriptional repressor BACH-1 via heme binding^27,28^. Similar effects were observed in HCT116 cells, although Nrf2 stabilization and particularly HO-1 expression were lower as in HCEC (Fig. 4f). Taken together, Nrf2 and its downstream target HO-1 are rapidly induced by hemin, which preferentially occurred in HCEC.

### Chemical inhibition or genetic ablation of HO-1 potentiates hemin-triggered ROS and oxidative DNA damage

**Fig. 5:**
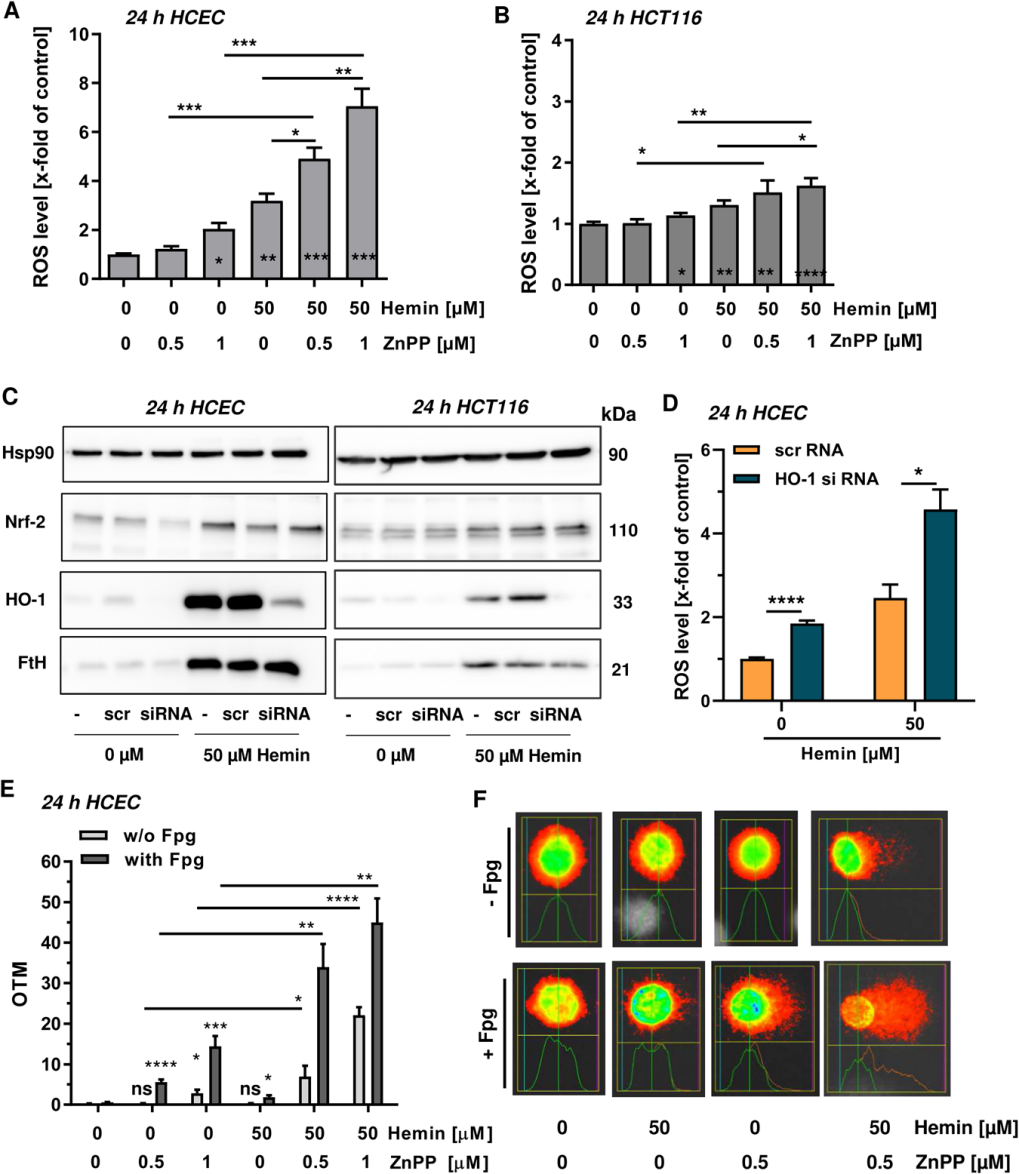
Influence of HO-1 on hemin-triggered ROS and oxidative DNA damage in HCEC and CRC cells. **a** HCEC were incubated with hemin (0 or 50 µM) in the absence or presence of the HO-1 inhibitor zinc protoporphyrin (ZnPP; 0.5 or 1 µM) for 24 h. Cells were stained with the CM-H2DCFDA dye and levels of reactive oxygen species (ROS) were assessed by flow cytometry. Data are presented as mean + SEM (*n* ≥ 3). *p < 0.05; **p < 0.01; ***p < 0.001. **b** HCT116 cells were treated and analyzed as described above. Data are given as mean + SEM (*n* = 4). *p < 0.05; **p < 0.01; ****p < 0.0001. **c** HCEC and HCT116 cells were transiently transfected with scrambled (scr) or HO-1-specific siRNA. 24 h after transfection, cells were treated with hemin and incubated for another 24 h. Whole-cell extracts were analyzed by SDS-PAGE and immunoblot detection of Nrf2 and FtH. Hsp90 served as loading control, whereas HO-1 was visualized to confirm knockdown. **d** Knockdown of HO-1 in HCEC followed by hemin exposure for 24 h and analysis of ROS formation. ROS were measured by flow cytometry as stated above. Data are given as mean + SEM (*n* = 4). *p < 0.05; ****p < 0.0001. **e** HCEC were treated with hemin (0 or 50 µM) in the absence or presence of the HO-1 inhibitor ZnPP (ZnPP; 0.5 or 1 µM) for 24 h. Cells were then subjected to the alkaline Comet assay with or without Fpg. OTM, olive tail moment. Data are given as mean + SEM (*n* = 5). Ns: *p* > 0.05; *p < 0.05; **p < 0.01; ***p < 0.001, ****p < 0.0001. **f** Representative pictures of the data shown in **e**.

In order to study the role of HO-1 in cytoprotection and to figure out, which intracellular iron forms (hemin or inorganic iron produced by heme catabolism) are crucial for the geno- and cytotoxicity, we made use of the HO-1 inhibitor zinc protoporphyrin (ZnPP) that blocks heme breakdown^29^. First, hemin-triggered ROS formation was analyzed in HCEC in the presence or absence of ZnPP. The HO-1 inhibitor itself slightly elevated ROS levels, while 50 µM hemin resulted in a pronounced increase in ROS (Fig. 5a and Supplementary Fig. S6A). Intriguingly, the combination of ZnPP and hemin strongly potentiated ROS formation, indicating that hemin itself is catalyzing ROS production (Fig. 5a and Supplementary Fig. S6A). Using the same experimental setup, almost no effects were observed in HCT116 cells (Fig. 5b and Supplementary Fig. S6B). To corroborate these results, HO-1 was downregulated by siRNA in both cell types (Fig. 5c). Treatment of HCEC with 50 µM hemin caused a strong induction of HO-1 in untransfected cells or cells transfected with scrambled RNA, while HCEC with siRNA mediated HO-1 knockdown showed only very little induction (Fig. 5c, left panel). As expected, hemin provoked a robust upregulation of both Nrf2 and its downstream target FtH in HCEC independent of the HO-1 knockdown. In HCT116 cells, HO-1 knockdown prevented its induction by hemin, similar to HCEC (Fig. 5c right panel). The genetic abrogation of HO-1 had no impact on Nrf2 and FtH induction following hemin treatment, both of which were however not as prominent as in HCEC (Fig. 5c). HO-1 knockdown moderately elevated basal ROS levels and strongly promoted ROS generation in HCEC upon hemin treatment (Fig. 5d and Supplementary Fig. S7A, B), which is in agreement with the results obtained with ZnPP (Fig. 5a). In HCT116 cells, siRNA mediated downregulation of HO-1 slightly increased basal ROS levels and potentiated ROS formation only in the presence of 200 µM hemin (Fig. S7C, D), consistent with the findings obtained for ZnPP treatment (Fig. 5b). Afterwards, induction of DNA damage was studied using the alkaline Comet assay with Fpg modification. Single treatment of HCEC with the inhibitor already increased the level of oxidative DNA damage, which was particularly obvious at a dose of 1 µM ZnPP (Fig. 5e). Incubation with 50 µM hemin caused only little DNA damage after 24 h as seen before (Fig. 5e and Supplementary Fig. S3). In the presence of the HO-1 inhibitor, both DNA strand breaks and oxidative DNA lesions were massively elevated as compared to the single treatments (Fig. 5e, f). Altogether, these findings strongly suggest that hemin, but not its cellular degradation product inorganic iron, is responsible for ROS generation and DNA damage induction.

### Inhibition or knockdown of HO-1 promotes hemin-triggered cytotoxicity

**Fig. 6:**
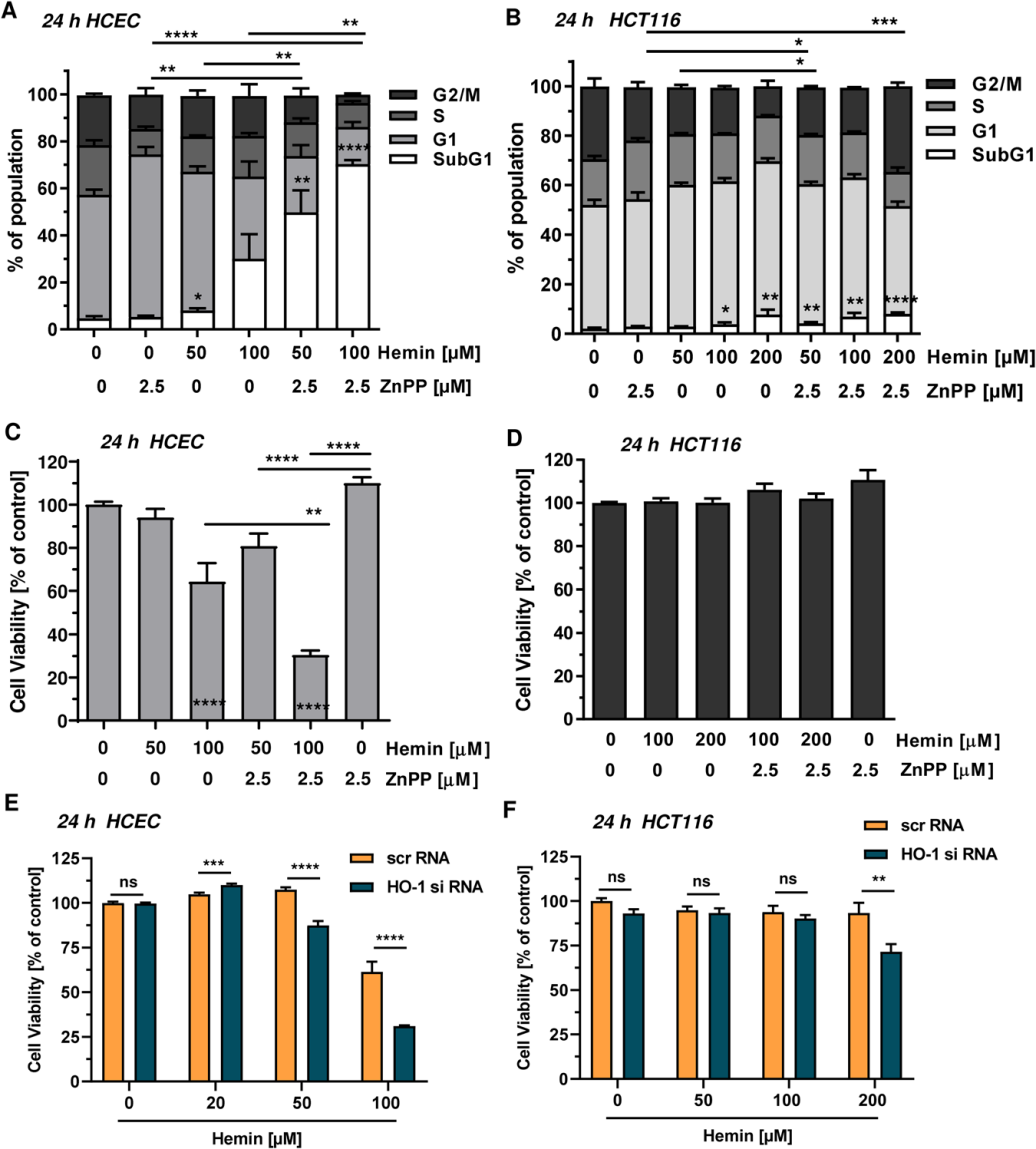
Influence of HO-1 on hemin-triggered cell cycle distribution and cytotoxicity in HCEC and CRC cells. **a** HCEC were treated with hemin (0–100 µM) in the absence or presence of the HO-1 inhibitor zinc protoporphyrin (ZnPP; 2.5 µM) for 24 h and collected for cell cycle analysis by flow cytometry. Data were evaluated by BD FACS Diva software and are shown as mean + SEM (*n* = 4). Statistical evaluation performed for subG1 population (white bars). *p < 0.05; **p < 0.01; ****p < 0.0001. **b** HCT116 cells were treated and analyzed as described above. Data are given as mean + SEM (*n* ≥ 3). *p < 0.05; **p < 0.01; ***p < 0.001; ****p < 0.0001. **c** HCEC were treated as described in **a** and cell viability was assessed using the MTS assay. Data are shown as mean + SEM (*n* ≥ 2, triplicates). **p < 0.01; ****p < 0.0001 **d** HCT116 cells were treated and analyzed as described in **a**. Data are given as mean + SEM (*n* = 2, triplicates). **e** HCEC were transiently transfected with scrambled (scr) or HO-1-specific siRNA. 24 h following transfection, cells were treated with increasing hemin concentrations for another 24 h. Cell viability was determined by the MTS assay. Data are shown as mean + SEM (*n* = 4, triplicates). Ns: *p* > 0.05; ***p < 0.001, ****p < 0.0001. **f** HCT116 cells were transiently transfected with scrRNA or HO-1 specific siRNA. Further treatment and analysis as described under **e**. Data are shown as mean + SEM (*n* ≥ 3, triplicates). Ns: *p* > 0.05; **p < 0.01.

**Fig. 7:**
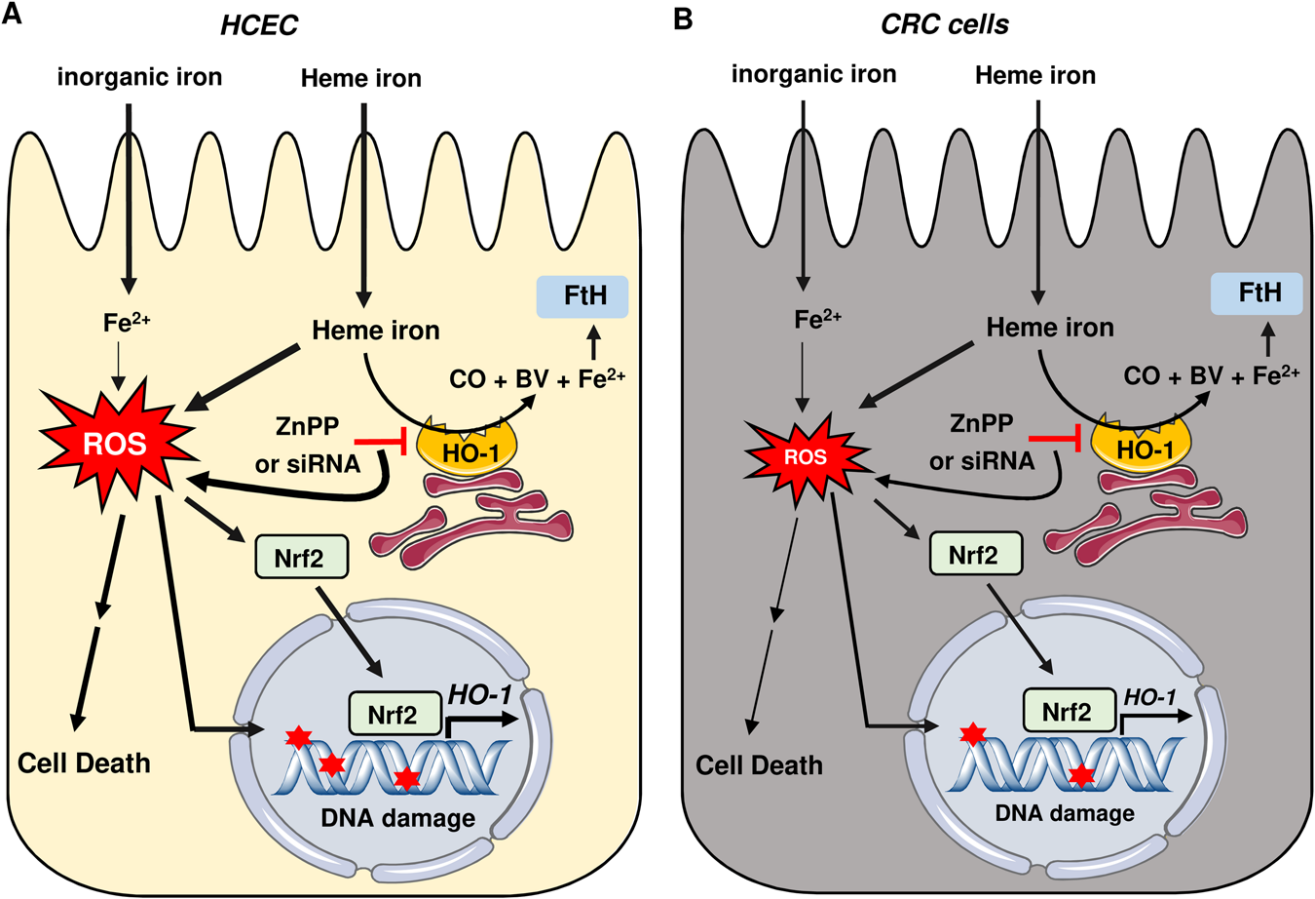
Model of heme-triggered DNA damage and cytotoxicity in HCEC versus CRC cells and role of HO-1. **a** Heme iron is taken up into HCEC, where it catalyzes the formation of reactive oxygen species (ROS) and induces oxidative DNA lesions as well as DNA strand breaks (indicated by red asterisks). This finally results in cytotoxicity, which is more prominent in HCEC than in CRC cell lines. In contrast, internalized inorganic iron causes little ROS production and DNA damage, and only slightly impairs cell viability. Heme-dependent ROS formation activates the transcription factor Nrf2, which shuttles from the cytoplasm to the nucleus, where it drives the transcription of its target genes such as heme oxygenase-1 (HO-1). This enzyme degrades heme to Fe^2+^, carbon monoxide (CO) and biliverdin (BV). Concomitant to HO-1 induction, ferritin heavy chain gene (FtH) is upregulated by hemin. Genetic abrogation of HO-1 by siRNA or its pharmacological inhibition by ZnPP potentiated heme-induced ROS, DNA damage and cell death, strongly suggesting that heme iron, and not its breakdown product Fe^2+^, initiates ROS formation and thus DNA damage induction. **b** Internalization of heme iron and inorganic iron in CRC cells. Similar to HCEC, inorganic iron causes little ROS production and DNA damage, and only slightly impairs viability in CRC cells. Hemin is taken up differentially into CRC cells (Caco-2 > HCT116), causing less ROS formation and oxidative DNA damage as in HCEC. Furthermore, CRC cells are in general more resistant against hemin-triggered cytotoxicity and HO-1 abrogation moderately affects cell survival in the presence of high heme concentrations. This figure was created using Servier Medical Art templates, which are licensed under a Creative Commons Attribution 3.0 Unported License; https://smart.servier.com.

Finally, we wished to know whether HO-1 inhibition by ZnPP affects cell cycle progression and viability in cells after hemin exposure. Hemin caused a dose-dependent increase in the subG1-population of HCEC, indicative of cell death (Fig. 6a and Supplementary Fig. S8A). HO-1 inhibition itself did not elevate the subG1-population as compared to the control, but increased the number of cells in G1-phase. Interestingly, combined treatment of HCEC with hemin and the HO-1 inhibitor strongly increased the subG1-population (Fig. 6a and Supplementary Fig. S8A). The cell cycle distribution of HCT116 cells was only significantly affected at a dose of 200 µM hemin (Fig. 6b and Supplementary Fig. S8B). Treatment of HCT116 with the HO-1 inhibitor resulted in a moderate increase of cells in S-phase at the expense of a reduced G2/M-phase (Fig. 6b and Supplementary Fig. S8B). In contrast to the findings in HCEC, HO-1 inhibition did not augment the subG1-population induced by high hemin concentrations in HCT116, but rather shifted cells from S-phase to G2/M-phase (200 µM hemin vs. 200 µM hemin + ZnPP). Subsequently, cell viability was assessed using the MTS assay in both HCEC and HCT116 cells. The HO-1 inhibitor itself slightly improved viability of HCEC, while treatment with 100 µM hemin caused a significant reduction in viability (Fig. 6c). Intriguingly, the presence of the HO-1 inhibitor potentiated the hemin-dependent loss of viability in HCEC, which is in agreement with the findings described above. This was further confirmed using the ATP assay as endpoint, revealing strongly enhanced cytotoxicity if HO-1 dependent degradation of hemin is blocked using ZnPP (Supplementary Fig. S9A, B). Next, HO-1 expression was silenced by siRNA in both HCEC and HCT116 cells. Knockdown of HO-1 potentiated hemin-induced cytotoxicity in HCEC (Fig. 6e), which is in line with the experiments using the HO-1 inhibitor ZnPP (Fig 6a, c). In contrast to that, knockdown of HO-1 in HCT116 cells only moderately affected viability at 200 µM hemin (Fig. 6f), confirming the results obtained with ZnPP treatment (Fig. 6b, d). In summary, the results highlight the cytoprotective role of HO-1 following hemin exposure and reveal that hemin, but not its breakdown product inorganic iron, causes cytotoxicity. The major findings have been summarized in a scheme presented in Fig. 7.

## Discussion

The present work compared the genotoxic and cytotoxic effects of hemin and inorganic iron side-by-side in HCEC versus CRC cell lines. First, we demostrated that hemin (≥20 µM) caused substantial ROS formation in a concentration-dependent manner in both HCEC and CRC cells, whereas inorganic iron had only very little or no effects. Notably, this held true for both ferric and ferrous iron. In line with the latter finding, ferric iron added as ferric nitrilotriacetate (Fe-NTA) promoted ROS formation in HT29 CRC cells only at a very high dose of 1000 µM^30^. Similarly, treatment of the hepatic cell line HH4 with ferric ammonium citrate resulted in signifcantly increased ROS levels only at concentrations above 1000 µM, while incubation of cells with 100 µM ferric ammonium citrate or ferric chloride did not augment basal ROS levels^31,32^. In support of our results, another study also reported ROS generation in HCT116 and RKO cells treated with 100 µM hemin^33^. Furthermore, we showed that hemin, in contrast to inorganic iron, caused DNA strand breaks as well as oxidative DNA damage (i.e., 8-oxoG) in HCEC and CRC cells. DNA strand break induction was previously observed in other CRC cell lines (i.e., Caco-2 and HT29) and in primary colonocytes exposed to hemin at concentrations ≥100 µM^14,34,35^. DNA strand break formation was also detected in isolated colonocytes from mice fed with red meat^36^. To the best of our knowledge, the present study is the first that demonstrated hemin mediated formation of 8-oxoG in HCEC or CRC cells. Whether or not this occurs also in vivo following dietary heme intake has to be elucidated in future studies. In agreement with the lack of genotoxicity for inorganic iron (Fe^2+^ and Fe^3+^) observed herein, ferric iron (applied as Fe-NTA) was reported to cause DNA strand breaks only at very high doses (≥500 µM) in colon adenoma and primary colon cells^37^. This is further supported by the finding that dietary ferric citrate caused significantly lower levels of the DNA strand break marker γ-H2AX in the colon mucosa of B6/J mice as compared to a diet with 2.5 % hemoglobin^18^.

It has recently been calculated that consumption of 100–500 g red meat (beef) will result in luminal heme concentrations ranging from 17–85 µM in humans^35^. Furthermore, data are available from rodent studies, in which the animals were fed with various dietary heme sources (e.g. beef, black pudding, hemin, hemoglobin, processed meat). Heme concentrations between 19–1097 µM were determined in fecal water obtained from the animals^16,38,39^, indicating that high heme concentrations can occur in the large intestine following its dietary uptake. Overall, the heme concentrations used in our study (20–200 µM) are in the range of those expected in humans and measured in fecal water from animal feeding studies.

Since ROS formation and DNA damage can both trigger cytotoxic effects, this issue was addressed using different endpoints. On the one hand, our experiments revealed that inorganic iron did neither affect cell cycle distribution nor caused cytotoxicity in HCEC, HCT116 cells and murine intestinal organoids, although being efficiently taken up particularly in HCEC as attested by ICP-MS analysis of the cellular iron content. This is pretty well in line with the observed lack of substantial ROS formation and genotoxicity in our study. On the other hand, hemin was demonstrated to be cytotoxic in a dose-dependent manner in non-malignant HCEC and murine intestinal organoids. Interestingly, CRC cell lines were generally more resistant in the order LS174T > HCT116 > Caco-2 ≈ RKO. One explanation for these findings could be differences in the cellular uptake efficiency and kinetics of both inorganic iron and heme iron. Ferric iron needs to be reduced for its intestinal uptake, which is catalyzed by ferric reductases such as the duodenal cytochrome B together with reducing compounds (e.g., dietary vitamin C)^40^. Ferrous iron is then mainly internalized by the divalent metal transporter 1 (DMT1)^41^. Screening of the used cell lines by western blot analysis revealed differential DMT1 expression (Supplementary Fig. S5F), with the highest expression levels in Caco-2 and HCT116 cells. HCEC displayed rather low DMT1 expression, but internalization of inorganic iron was better than in HCT116 and Caco-2 cells. This may be attributable to the required supplementation of the HCEC cell culture medium with transferrin (2 µg/ml ≈ 25 µM), which promotes the uptake of ferric iron by receptor-mediated endocytosis. Two pathways were described for the internalization of heme into enterocytes. One pathway seems to involve receptor-mediated endocytosis mediated by a still unknown protein^42^. Moreover, an intestinal heme transporter called HCP-1 was identified^4^, which was also discovered as an intestinal folate transporter in a subsequent study^43^. Western blot detection of HCP-1 in the cell lines used in this study revealed high levels in HCEC and RKO cells, whereas HCT116 and Caco-2 cells displayed only low expression (Supplementary Fig. S4F). Importantly, total cellular iron levels were determined by ICP-MS and showed higher levels in HCEC than in HCT116 cells following incubation with hemin, which could thus be responsibe for the stronger cytotoxic effects observed in HCEC. However, hemin exerted a weaker cytotoxicity in Caco-2 cells despite even higher iron levels as compared to HCEC. Furthermore, RKO cells displayed a moderate sensitivity towards hemin similar to Caco-2 cells, but expressed much higher HCP-1 levels. These findings indicate an involvement of another, HCP-1 independent heme uptake mechanism and highlights the need to measure cellular iron concentrations as done in the present study. It should also be mentioned that intracellular iron levels are further controlled by efflux transporters as described elsewhere^7^.

As the cellular iron levels not directly correlate with the observed cytotoxicity in HCEC and the CRC cell lines, other factors likely contribute to the resistance of CRC cells towards hemin. The tumor suppressor protein p53, which is activated by genotoxic stress and can trigger cell death, could be considered here given that it is frequently mutated in CRC cells^44^. However, both the resistant HCT116 and LS174T CRC cells as well as the sensitive HCEC express wildtype p53^45,46^, thus rather excluding this possibility. Previous studies showed an increased cytotoxicity of fecal water from hemin or hemoglobin fed rodents and its constitiuent 4-HNE in non-transformed murine colonocytes with wildtype APC^17,18^. Intriguingly, murine colonocytes harboring a mutated APC allel (APC^Min/+^) were more resistant, which could be due to the higher expression of enzymes implicated in HNE detoxification^19^. Among the human CRC cell lines tested here, LS174T, HCT116 and RKO cells express wildtype APC, while Caco-2 cells bear APC mutations^47,48^. Thus, no correlation was observed in these cell lines between their sensitivity to hemin and their APC status. Consistent with this finding, murine APC^+/+^ and APC^Min/+^ colonocytes displayed similar reduction in viability after treatment with hemin^18^.

Furthermore, we demonstrated that hemin causes Nrf2 stabilization and its translocation into the nucleus of HCEC, which was followed by a robust induction of cytosolic HO-1. In contrast, inorganic iron had little effect on Nrf2 levels and failed to induce HO-1, which goes along with the absence of oxidative stress. A similar upregulation of Nrf2 was detected in murine colonocytes exposed to fecal water from hemoglobin- and beef-fed rats, which was attenuated by former trapping of reactive aldehydes present in fecal water^20^. Further evidence for Nrf2 activation came from a feeding study in mice, which received hemin or ferric citrate as control for up to 14 days. Hemin-fed mice displayed a fast and sustained upregulation of several Nrf2 target genes including *catalase*, *glutathione S-transferase* and *heme oxygenase 1* (*Hmox-1*)^11,49^. Altogether, these findings emphasize the important role of Nrf2 signaling in response to oxidative stress triggered by heme iron. Furthermore, we showed that hemin also stimulated FtH expression in a dose-dependent manner, which paralleled the induction of HO-1. FtH is a known Nrf2 target gene^50^ and is also controlled on the translational level by iron-regulatory proteins in a time-dependent manner^51^. Nrf2 is responsible for basal Fth1 expression and induces ferritin expression upon oxidative stress^52^. In line with our findings, hemin was reported to upregulate the expression of FtH in K562 human erythroleukemic cells^53^.

To elucidate the role of HO-1 we used ZnPP as competitive HO-1 inhibitor and, in addition, performed a siRNA-mediated knockdown of HO-1, thereby revealing strongly increased ROS generation and DNA damage induction (*i.e*. DNA strand breaks, 8-oxoG) in HCEC. This is very likely attributable to the blocked heme catabolism and the concomitant accumulation of intracellular free heme. Genetic or chemical abrogation of HO-1 in HCEC potentiated hemin-dependent cytotoxicity as evidenced by cell viability assays and analysis of cell cycle distribution. Notably, HCT116 cells were almost unaffected after incubation with comparable doses of hemin and ZnPP. Our findings clearly demonstrate a cytoprotective role for HO-1 in normal human colonocytes and further indicate that free heme, but not free iron generated following heme catabolism, is mainly responsible for the genotoxic and cytotoxic effects. In this regard, it is important to mention that free iron produced from heme by HO-1 is quickly neutralized by diverse mechanisms, such as the upregulation of FtH as described above and gene induction of iron efflux pumps^24^. Furthermore, HO-1 mediated degradation of heme generates biliverdin and the gasotransmitter CO^6^. Biliverdin is transformed into bilirubin by biliverdin reductase, which is known to possess antioxidative activity^54^. CO is also involved in the cytoprotective effects exerted by HO-1^24^. Interestingly, HO-1 induction and CO generation were reported to promote the repair of DNA double strand breaks presumably via ATM as shown in *Hmox-1*^−/−^ mice and fibroblasts derived thereof^55^. These mechanisms could further add to the observed increase in ROS formation and DNA strand break induction in HCEC treated with hemin under HO-1 inhibition. Finally, the cytoprotective function of HO-1 demonstrated here in human colonocytes is consistent with the high ROS levels and decreased survival of *Hmox-1*^−^^/−^ embryonic fibroblasts exposed to hemin^56^.

In conclusion, our study provided evidence that free heme iron, but not its breakdown product inorganic iron, plays a pivotal role in ROS formation and DNA damage induction in human colonocytes. HO-1 was further revealed to confer protection against the detrimental effects of hemin. Interestingly, CRC cell lines were generally more resistant to the cytotoxic effects of hemin than normal colonocytes, which might favor the outgrowth of neoplastic cells in vivo and could thereby promote intestinal carcinogenesis.

## Material and methods

### Cell culture and treatment

Non-transformed human colon epithelial cells (HCEC 1CT) were kindly provided by Prof. Jerry W. Shay (Department of Cell Biology, UT Southwestern Medical Center, Dallas, USA). Cells were isolated from normal human colonic biopsies and immortalized by CDK4- and hTERT-transfection as described^46^. HCEC were grown in a nitrogen incubator with reduced oxygen levels (7% O_2_) and 5% CO_2_ at 37 °C in 4:1 DMEM GlutaMax/Medium 199 (ThermoFisher Scientific, Darmstadt, Germany). The medium was supplemented with 2% cosmic calf serum (Hyclone, GE Healthcare, Hamburg, Germany), 25 ng/ml human epidermal growth factor, 1 µg/ml hydrocortisone, 10 µg/ml insulin from bovine pancreas, 2 µg/ml transferrin, 5 nM sodium selenite and 50 µg/ml gentamycin sulfate (all from Sigma, Schnelldorf, Germany).

HCT116 cells were generously provided by Prof. Bert Vogelstein (John Hopkins University, Baltimore, USA) and obtained from the Core Cell Center (John Hopkins University). RKO cells were a kind gift of Prof. Oliver H. Krämer (Institute of Toxicology, University Medical Center, Mainz, Germany). RKO and HCT116 cells were cultured in DMEM containing 10 % fetal calf serum (FCS) (PanBiotech Aidenbach, Germany) and 1% penicillin/streptomycin (p/s). Caco-2 cells were purchased from CLS Cell Lines Service (Eppelheim, Germany) and maintained in MEM with 10% FCS, 1% p/s and nonessential amino acids. LS174T cells were a kind gift of Prof. Thomas Brunner (University of Konstanz, Konstanz, Germany) and cultured in IMDM medium (Pan Biotech, Aidenbach, Germany) with 10% FCS and 1% p/s. CRC cell lines were maintained at 37 °C in a humidified atmosphere of 5% CO_2_ under standard culture conditions (∼20% O_2_). For selected experiments, CRC cells were cultured under hypoxic conditions (7% O_2_) in the nitrogen incubator. Cell culture medium and supplements were obtained from Life Technologies (Darmstadt, Germany) unless stated otherwise. Cell lines were mycoplasma negative as demonstrated by PCR and immunofluorescence microscopy with nuclear staining. All cell lines were authenticated as described previously^57,58^.

Hemin (Fe^3+^ protoporphyrin IX chloride; Sigma, Schnelldorf, Germany) was dissolved in 20 mM sodium hydroxide (NaOH), while ferric chloride (FeCl_3_) and ferrous sulfate (FeSO_4_), both purchased at Sigma, were dissolved in water to obtain stock concentrations of 1 mM each. Zinc protoporphyrin (ZnPP; Enzo Life Science, Farmingdale, USA) was dissolved in DMSO at a final concentration of 5 mM.

### Transient transfection with siRNA tageting HO-1

Knockdown of HO-1 was performed using siGENOME SMARTpool siRNA from Dharmacon (Lafayette, USA). Cells grown in 6 well or 96 well plates were transfected at 40% confluency with 20 nM siRNA using Lipofectamine^®^ RNAimax (Invitrogen, Darmstadt, Germany). Scrambled, non-sense siRNA (20 nM; Dharmacon, Lafayette, USA) was included as negative control. 24 h after transfection, cells were treated with hemin for different incubation periods and analyzed as indicated. HO-1 knockdown was checked by SDS-PAGE and western blot detection.

### Generation of murine intestinal organoids and assessment of viability

Isolation of intestinal crypts, ex vivo culture and expansion to intestinal organoids were essentially performed as described^59^. C57BL6/J mice (8–12 weeks) were sacrificed followed by the preparation of the small intestine. The organ was opened longitudinally and cut into 1–2 cm pieces after villi had been scraped off with a microscope slide. The tissue slices were washed with ice-cold PBS and then incubated in PBS containing 2 mM EDTA for 30 min at 4 °C on a rotator. Subsequently, PBS/EDTA was removed and replaced by PBS. After gentle shaking, the supernatant was monitored by light microscopy for residual villi and containing crypts. This process was repeated until the villi/crypt ratio in the supernatant was optimal, showing the highest number of intact crypts. The suspension was then filtered through a 70 µm cell strainer (BD Biosciences, Heidelberg, Germany) to remove residual villi, pelleted by centrifugation and crypts were resuspended in PBS for counting. After another centrifugation step, crypts were resuspendend in Matrigel (BD Biosciences) at the desired density (typically 100–300 crypts per 8 µl of Matrigel). Crypts were seeded into a flat bottom 96 well plate by applying a droplet of 8 µl Matrigel to each well, which was allowed to polymerize at 37 °C for 30 min. Afterwards, 80 µl of complete crypt culture medium was added consisting of advanced DMEM/F12, 10 mM HEPES, 100 U/ml penicillin, 100 mg/ml streptomycin, 20 mg/ml nystatin, 1 mM N-acetyl cysteine (all from Sigma, Schnelldorf, Germany), 0.1% BSA (Carl Roth, Karlsruhe, Germany), 2 mM l-glutamine, 1x B27 supplement, 1x N2 supplement (all from Life Technologies, Darmstadt, Germany), 50 ng/ml murine EGF, 100 ng/ml murine Noggin and 500 ng/ml human R-spondin-1 (all from Peprotech, Hamburg, Germany). Intestinal organoids were maintained at 37 °C and 5% CO_2_ in the incubator for 3 days. Organoids were then exposed to increasing concentrations of hemin or ferric chloride (0–100 µM) for 24 h. Viability was determined using the MTT assay. To this end, MTT solution was added to the organoid culture at a final concentration of 500 µg/ml. After an incubation for 1 h, the medium was discarded and 20 µl of 2% SDS solution was added in order to solubilize the Matrigel. After another 1 h of incubation, the solution was discarded and the reduced MTT was solubilized with 80 µl DMSO for 1 h. Absorbance at 562 nm was determined using a microplate reader (Sunrise Tecan Reader, Crailsheim, Germany). Solvent treated organoids were defined as 100% viable.

### Determination of cell viability by ATP and MTS assay

Viability of CRC cells and HCEC was determined using the Cell Titer 96® AQueous One Solution Cell Proliferation Assay (Promega, Mannheim, Germay) as reported^58^. Cells (HCT116: 5 × 10^3^/well; Caco-2: 5 × 10^3^/well; LS174T: 5 × 10^3^/well, RKO: 5 × 10^3^/well and HCEC: 1 × 10^3^/well) were grown overnight and then treated with increasing concentrations of hemin or iron chloride (0–200 µM as indicated). NaOH or H_2_O served as respective solvent controls. After 72 h, viability was measured using a microplate reader (Sunrise Tecan Reader, Crailsheim, Germany) according to the manufacturer’s instructions. To analyze the effects of hemin in the absence or presence of the HO-1 inhibitor ZnPP, HCT116 cells (24 h: 1.5 × 10^4^ cells/well; 72 h: 5 × 10^3^ cells/well) or HCEC (24 h: 2.5 × 10^3^/well; 72 h: 1 × 10^3^/well) were grown in white 96-well plates overnight. Cells were then preincubated with ZnPP (0–2.5 µM) for 1 h followed by the addition of hemin (0–100 µM). Viability was assessed after 24 or 72 h using the MTS assay as described above. Furthermore, viability was determined with the CellTiter-Glo®Luminescent Cell Viability Assay (Promega, Mannheim, Germany), which measures the cellular ATP level. The assay was performed according to the manufacturer’s instructions with a MicroLumat Plus 96-well plate reader (Berthold, Bad Wildbad, Germany).

### ICP-MS analysis to determine total cellular iron levels

Caco-2 cells (1 × 10^5^), HCT116 cells (5 ×10^5^) or HCEC (2 × 10^5^) were grown in 6 cm dishes and treated with hemin or ferric chloride for 0.25, 1, 8 and 24 h. Cells were trypsinized and harvested by centrifugation. The pellets were then digested in 0.5 ml H_2_O_2_/HNO_3_ (1:1) overnight at 95 °C. The residue was dissolved in 1.5 ml 10% HNO_3_ with 0.5 µg/l Rh (Merck, Darmstadt, Germany) as internal standard and diluted if required. Quantification was performed with an external calibration curve ranging from 0.5–100 µg/l Fe (Spetec, Erding, Germany). The measurements were conducted with a tandem mass spectrometer (8800 triple quadrupole ICP-MS, Agilent Technologies, Böblingen, Germany) equipped with two quadrupols (Q1 and Q2) as well as a collision and reaction cell with O_2_ (0.3 ml/min) as collision gas. Quantification was carried out directly using the ^56^Fe^+^ ion (Q1 and Q2 mass-to-charge (m/z) ratio 56) or indirectly using the ^56^Fe^16^O^+^ ion (Q1 m/z 56 and Q2 m/z 72).

### SDS–PAGE and Western blot

HCT116 cells (3 × 10^5^) or HCEC (2 × 10^5^) were seeded in 3.5 cm dishes and allowed to adhere overnight. Following treatment with hemin or ferric chloride as indicated, cells were harvested in 1× Lämmli loading buffer. Western blot analysis was essentially performed as described^57^. Equal protein amounts were separated by SDS-PAGE followed by transfer onto a nitrocellulose membrane (Amersham™ Protran®, GE Healthcare, Freiburg, Germany) with a wet blot chamber (BioRad, München, Germany). Membranes were then blocked with 5% nonfat dry milk (Carl Roth) in Tris-buffered saline (TBS)/0.1% Tween-20 for 1 h at RT. Primary antibody incubation was conducted overnight at 4 °C followed by three washing steps (3 × 5 min) in TBS/0.1% Tween-20. Membranes were then incubated with appropriate secondary antibodies for at least 1 h at RT. After 3 × 5 min washing, proteins were detected using Western Lightning® Plus-ECL (Perkin Elmer, Rodgau, Germany). The following primary antibodies anti-ferritin heavy chain gene (FtH; mouse monoclonal, B-12; no. sc-376594), anti-heat shock protein 90 (Hsp90) *α/β* (mouse monoclonal; no. sc-13119), anti-heme carrier protein-1 (HCP-1; B-4; mouse monoclonal; no. sc-393460) were all purchased from Santa Cruz Biotechnology, Heidelberg, Germany. The primary antibodies anti-divalent metal transporter-1 (DMT1; rabbit polyclonal; no. GTX64686), anti-heme oxygenase-1 (HO-1; rabbit polyclonal; no. GTX101147) and anti-nuclear factor E2-related factor (Nrf2; rabbit monoclonal; no. GTX103322) were obtained from GeneTex, Irvine, California, USA. Secondary antibodies conjugated with horseradish-peroxidase were from Santa Cruz (anti-mouse) and Cell Signaling (anti-rabbit).

### Measurement of ROS formation by flow cytometry

HCT116 cells (3 × 10^5^/well), Caco-2 cells (2 × 10^5^/well) and HCEC (2 × 10^5^/well) were grown overnight in six-well plates. Cells were treated with increasing doses of hemin or ferric chloride (0–200 µM) for up to 24 h. Incubation with 200 µM H_2_O_2_ (Merck, Darmstadt, Germany) for 20 min in PBS served as positive control. Levels of reactive oxygen species (ROS) were determined as reported^60^. Briefly, cells were rinsed twice with pre-warmed PBS and then were loaded with 2.5 µM CM-H2DCFDA (Invitrogen, Darmstadt, Germany) for 30 min at 37 °C using phenol red- and serum-free medium. Following a washing step with PBS, cells were harvested using Trypsin/EDTA, pelleted by centrifugation and resuspended in PBS. Finally, cells were analyzed by flow cytometry using a BD FACSCanto II (BD Biosciences, Heidelberg, Germany) and evaluated using BD FACSDiva software.

### Assessment of cell cycle distribution by flow cytometry

Cell cycle analysis was performed as described^61^. HCT116 cells (5 × 10^5^) and HCEC (3 × 10^5^) were grown overnight in 6 cm dishes and then exposed to increasing doses of hemin or ferric chloride in the presence or absence of ZnPP as indicated. After 24 h of incubation, cells were harvested and washed twice in PBS. Following ethanol precipitation at −20 °C for 1 h, the pellet was resuspended in PBS containing RNase A (20 μg/ml) and incubated for 1 h at RT. Subsequently, propidium iodide (PI; Sigma) was added to a final concentration of 10 μg/ml, and cells were analyzed for DNA content by flow cytometry using BD FACSCanto II (BD Biosciences). Cell cycle distribution was analyzed with BD FACSDiva software.

### Detection of DNA damage by the Comet assay

HCT116 cells (2.5 × 10^5^) and HCEC (1.5 × 10^5^) were seeded in 3.5 cm dishes and grown overnight. Cells were then incubated with increasing concentrations of hemin or ferric chloride for 2 or 24 h as stated. As positive control, the cells were treated with 50 µM tert-butyl hydroperoxide (t-BOOH; Sigma) for 20 min. Cells were then harvested and processed for the alkaline Comet assay as described^62,63^. Cells embedded in 0.5% low melting point agarose were transferred onto a slide pre-coated with agarose. The slides were incubated for 1 h in lysis buffer consisting of 2.5 M sodium chloride (NaCl), 100 mM ethyldiamine tetraacetic acid (EDTA), 1% Triton X-100 and 10 mM Tris pH 10. As a next step, DNA unwinding was conducted in electrophoresis buffer (300 mM NaOH, 1 mM EDTA pH 13) for 25 min at 4 °C. Samples were then subjected to electrophoresis for 15 min at 25 V and 300 mA followed by a neutralization step with 0.4 M Tris pH 7.5. After fixation in 100% ethanol, the air-dried samples were stained with 50 μg/ml PI (Sigma). Comets were analyzed by fluorescence microscopy using an Olympus BX50 microscope equipped with a ColorView camera (Olympus, Münster, Germany). In each experiment, at least 50 cells were scored using Comet IV software (Perceptive Instruments Ltd., Bury St Edmunds, UK).

To detect oxidative DNA damage (i.e., 8-Oxoguanine), the Formamidopyrimidine-DNA glycosylase (Fpg)-modified alkaline Comet assay was used^64^. After cell lysis, the slides were incubated in a buffer containing Fpg (1 µg/ml), 40 mM HEPES, 0.1 M KCL, 0.5 mM EDTA and 0.2 mg/ml BSA, pH 8.0 for 37 min at 37 °C. Subsequently, the protocol was continued with the DNA unwinding step as described above.

### Confocal microscopy of fixed cells

HCEC (1 × 10^5^ cells) were seeded on coverslips in 6-well plates. After 24 h, HCEC were treated with increasing doses of hemin (0–100 µM) and incubated for 8 h. Immunofluorescence staining was performed as reported^65^. Upon fixation with methanol at −20 °C for 10 min, cells were washed twice with PBS and then incubated for 1 h with blocking solution consisting of 5% bovine serum albumin (BSA) in PBS with 0.3% Triton X-100. The samples were incubated with an anti-Nrf2 antibody (Genetex; diluted 1:500 in PBS/0.2% Triton X-100) or an anti-HO1 antibody (Genetex; diluted 1:1,000 in PBS/0.2% Triton X-100) overnight at 4 °C. After several washing steps, the samples were incubated with an appropriate Alexa488-coupled goat-anti-rabbit secondary antibody (Life Technologies; 1:400 in PBS plus 0.2% Triton X-100). Nuclei were finally counterstained using TO-PRO-3 (Life Technologies; 1:100 in PBS). Coverslips were mounted using VectaShield (Vector Labs, Burlingame, USA) and analyzed with a Zeiss Axio Observer.Z1 microscope equipped with a confocal LSM710 laser-scanning unit (Zeiss, Oberkochen, Germany). Pictures were analyzed and processed using Image J.

### Cell fractionation

Cells were treated as indicated and subjected to cell fractionation according to a protocol published recently^65^. Protein content of the cytoplasmic and nuclear fractions was determined by the Bradford assay. SDS–PAGE and Western blot analysis were conducted as described above.

### Statistics

Experiments were performed independently three times, except when otherwise stated. Representative experiments are displayed. Values are presented as means + standard error of the means (SEM). Data were analyzed for outliers with the Grubbs’ test using GraphPad Prism 7.0 software. Statistical analysis was then performed in GraphPad Prism using two-sided Student’s *t*-test and statistical significance was defined as *p* < 0.05.

## Supplementary information

Supplementary Figure Legends

Figure S1

Figure S2

Figure S3

Figure S4

Figure S5

Figure S6

Figure S7

Figure S8

Figure S9
