# Supplementary material for: Heme oxygenase 1 protects human colonocytes against ROS formation, oxidative DNA damage and cytotoxicity induced by heme iron, but not inorganic iron
Source: Cell Death Dis. 2020 Sep 23;11(9):787. doi: 10.1038/s41419-020-02950-8 (PMC7511955; doi:10.1038/s41419-020-02950-8)
Supplement: Supplementary file 1 — Supplementary Figure Legends [file 41419_2020_2950_MOESM1_ESM.docx]

**Supplementary Figure Legends**

**Figure S1: Time-dependent ROS formation in HCEC and CRC cells treated with hemin or inorganic iron. A** HCEC were treated with hemin or FeCl_3_ as indicated for 30 min, 2 h or 24 h. Subsequently, reactive oxygen species (ROS) formation were assessed by live cell staining and flow cytometry-based analysis. Representative histograms are depicted. **B** Representative histograms of ROS formation in HCT116 cells treated with hemin or FeCl_3_.

**Figure S2: Formation of ROS in HCEC and CRC cells exposed to hemin or inorganic iron under normoxic and hypoxic conditions.** HCEC were incubated for 2 (**A**) or 24 h (**B**) with hemin, FeCl_3_ or FeSO_4_ as indicated or 20 min with 200 µM H_2_O_2_ as positive control. Reactive oxygen species (ROS) levels were assessed by live cell staining and subsequent flow cytometry-based analysis. Data is presented as mean + SEM (n≥3). Ns: p>0.05; ***p<0.001; ****p<0.0001 (versus control). **C** Caco-2 cells were incubated for 24 h with hemin (50 µM or 200 µM) or FeCl_3_ (200 µM). Data were analyzed as described under A and B. Ns: p>0.05; **p<0.01; ***p<0.001 (versus control). **D** HCT116 cells were exposed to increasing hemin concentrations or FeCl3 for 24 h and ROS levels were determined by flow cytometry. Data are given as mean + SEM (n≥3). Ns: p>0.05; *p<0.05; **p<0.01 (versus control). **E** HCT116 cells were treated as indicated under hypoxic conditions (7 % O_2_ instead of 21 %). Formation of ROS was measured as described before. Data are given as mean + SEM (n=3). Ns: p>0.05; *p<0.05; **p<0.01 (versus respective control).

**Fig. S3: Induction of DNA strand breaks and oxidative DNA damage in HCEC and CRC cells by hemin versus inorganic iron. A** and **B** Cells were incubated with hemin (0 - 200 µM) or FeCl_3_ (200 µM) for 24 h. DNA strand break induction (A) and oxidative DNA damage (B) were determined with the alkaline Comet assay without or with FPG, respectively. OTM: olive tail moment. # denotes concentrations not tested due to expected pronounced cytotoxicity (HCEC) or weak effects (HCT116). Data are shown as mean + SEM (n≥3). Ns: p>0.05; *p<0.05; ***, p<0.001; ****, p< 0.0001 (versus respective control). **C** HCEC were treated with 200 µM FeSO_4_ for 24 h or 20 min with 50 µM tBOOH, as positive control. DNA strand break induction (light grey) and formation of oxidative DNA damage (dark grey) was determined by the alkaline Comet assay with or without Fpg modification. OTM: olive tail moment. Data are presented as mean + SEM (n≥3). Ns: p>0.05; *p<0.05; ****p<0.0001.

**Figure S4: Impact of heme iron and inorganic iron on the viability of CRC cell lines under normoxic and hypoxic condition and expression of iron transporters. A – D** Cells were exposed to increasing doses of hemin or FeCl_3_ for 72 h under normoxic (A and B) or hypoxic (C and D) conditions. Cell viability of LS174T (A), RKO (B), HCT116 (C) and Caco-2 (D) were determined using the MTS assay. Data are shown as mean + SEM (n≥3, triplicates). Ns: p>0.05; **p<0.01; ***p<0.001; ****p<0.0001 (versus respective control); n.d. not determined **E** HCEC were exposed to hemin (200 µM) or FeSO_4_ (200 µM) for 24 h. Cell viability was determined using the ATP assay. Data shown as mean + SEM (n=2, triplicates). Ns: p>0.05; ****p<0.0001 (versus control). **F** HCEC and different CRC cell lines were studied for DMT-1 and HCP-1 expression by Western blot analysis. Hsp90 served as loading control.

**Figure S5: Impact of hemin and inorganic iron on Nrf2 signaling in HCEC and CRC cells. A** HCEC were exposed to 50 µM hemin for 2 h. Cells were fixed, processed for Nrf2 staining and analyzed by confocal microscopy. Representative images are shown. Nrf2 is depicted in green and nuclei are shown in blue. Scale bar: 20 µm. **B** HCEC were treated with 10 µM hemin for 8 h. Cells were fixed, processed for HO-1 staining and analyzed by confocal microscopy. Representative images are shown. HO-1 is depicted in green and nuclei are shown in blue. Scale bar: 20 µm. **C** HCT116 and HCEC were exposed to increasing concentrations of hemin or FeCl_3_ for 8 h. Cells were harvested, lysed and subjected to Western blot analysis for heme oxygenase-1 (HO-1). Hsp90 was detected as loading control. **D** HCT116 cells were treated for 24 h with hemin, FeCl_3_ or FeSO_4_ as indicated. Cell lysates were subjected to SDS-PAGE followed by western blot analysis for HO-1 and ferritin heavy chain (FtH) expression. Hsp90 was visualized as loading control. **E** HCEC treated and analyzed as described under D.

**Figure S6: ROS formation upon HO-1 inhibition in hemin-treated HCEC and CRC cells.** HCEC (**A**) and HCT116 cells (**B**) were incubated with hemin (0 or 50 µM) in the absence or presence of the HO-1 inhibitor zinc protoporphyrin (ZnPP; 1 µM) for 24 h. Cells were stained with the CM-H_2_DCFDA dye and levels of reactive oxygen species (ROS) were assessed by flow cytometry. Shown are representative histograms.

**Figure S7: Impact of HO-1 on ROS formation in hemin-treated HCEC and CRC cells. A and B** HCEC were transiently transfected with scrambled (scr) or HO-1 specific siRNA. 24 h after transfection, cells were exposed to heme (0 and 50 µM) and incubated for 24 h. Cell morphology was assessed by phase contrast microscopy (A). Cells were then stained with the CM-H_2_DCFDA dye and levels of reactive oxygen species (ROS) were assessed by flow cytometry. Shown are representative histograms (B). **C** HCT116 cells treated and analyzed as described above. Representative histograms are displayed. **D** Quantitative analysis of ROS formation in HCT1116 by flow cytometry (n≥3). Ns: p>0.05; **p<0.01; ***p<0.001.

**Figure S8: Influence of HO-1 inhibition on hemin-treated cell cycle distribution in HCEC and CRC cells.** HCEC (**A**) and HCT116 cells (**B**) were incubated with hemin (0 or 50 µM) in the absence or presence of the HO-1 inhibitor zinc protoporphyrin (ZnPP; 2.5 µM) for 24 h. Cell cycle distribution was analyzed by flow cytometry and data were evaluated by BD FACS Diva software. Shown are representative histograms.

**Figure S9: Influence of HO-1 inhibition on hemin-treated cytotoxicity in HCEC.** HCEC were treated with increasing doses of hemin (0 – 100 µM) with or without the HO-1 inhibitor zinc protoporphyrin (ZnPP; 2.5 µM) for 24 h (**A**) or 72 h (**B**). Cell viability was determined using ATP assay. Data are depicted as mean + SEM (n≥2, triplicates). **p<0.01; ***p<0.001; ****p<0.0001.
