# Supplementary figures and images for: Heme oxygenase 1 protects human colonocytes against ROS formation, oxidative DNA damage and cytotoxicity induced by heme iron, but not inorganic iron
Source: Cell Death Dis. 2020 Sep 23;11(9):787. doi: 10.1038/s41419-020-02950-8 (PMC7511955; doi:10.1038/s41419-020-02950-8)

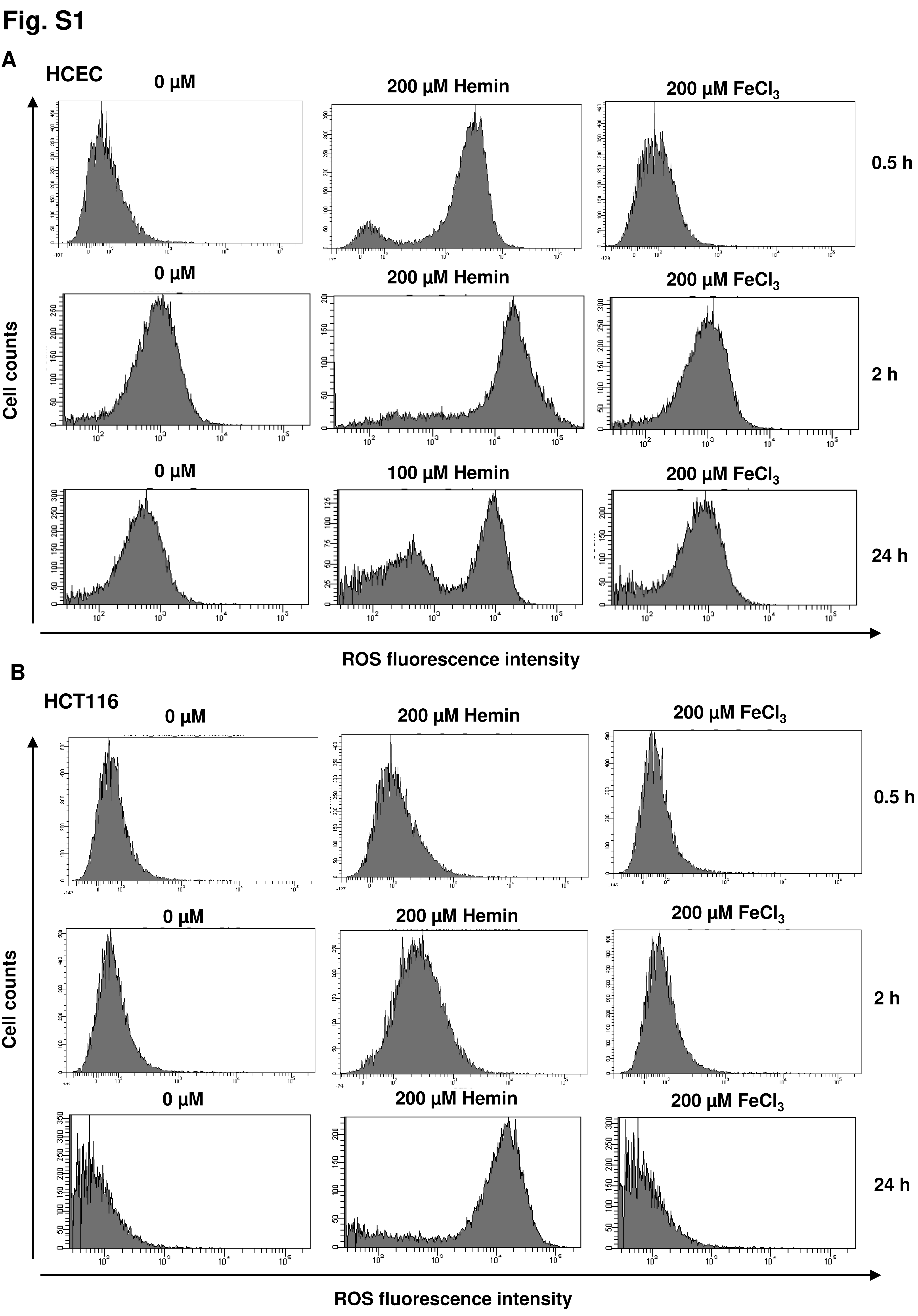

Supplement: Supplementary file 2 — Figure S1 [file 41419_2020_2950_MOESM2_ESM.tif]

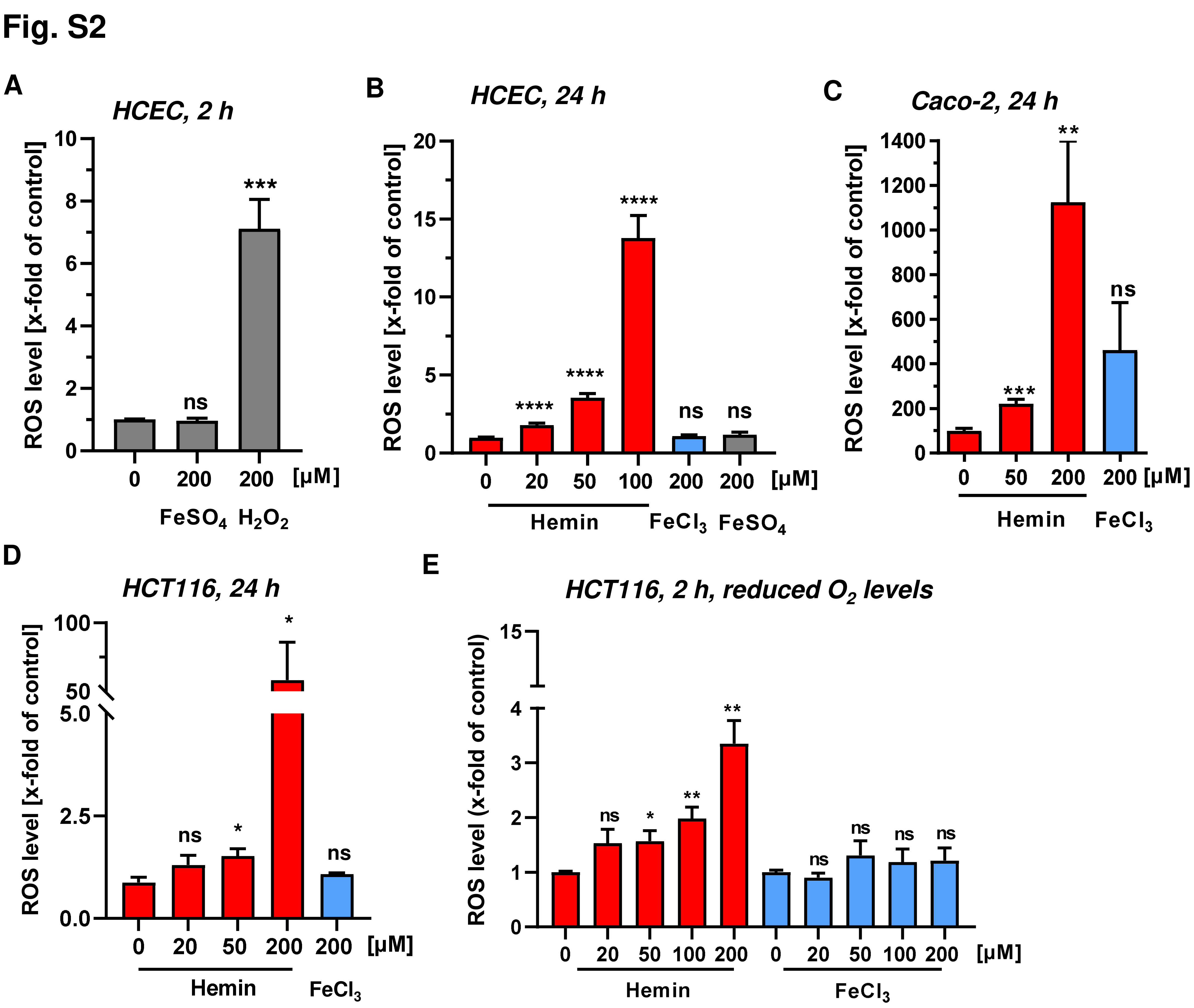

Supplement: Supplementary file 3 — Figure S2 [file 41419_2020_2950_MOESM3_ESM.tif]

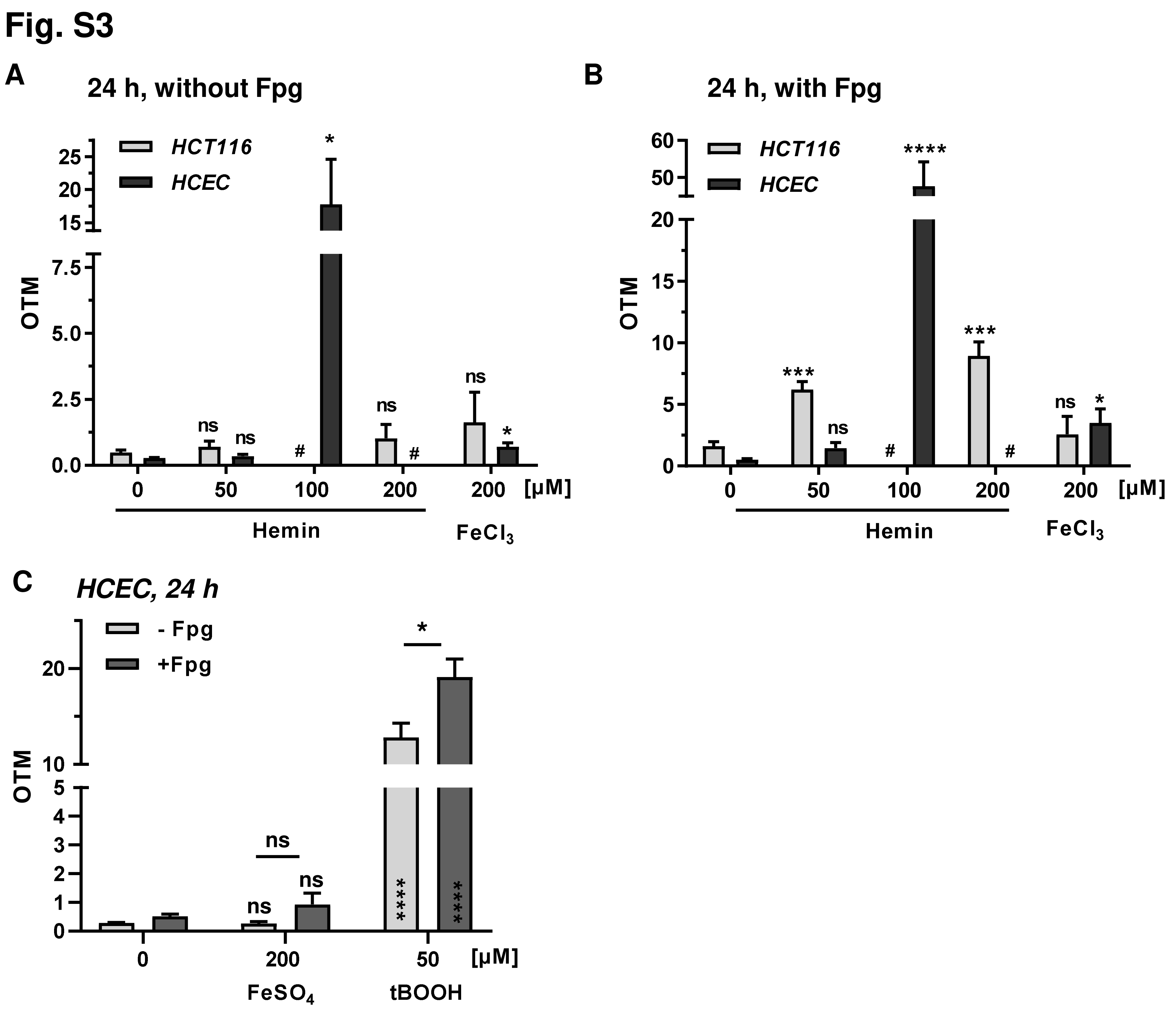

Supplement: Supplementary file 4 — Figure S3 [file 41419_2020_2950_MOESM4_ESM.tif]

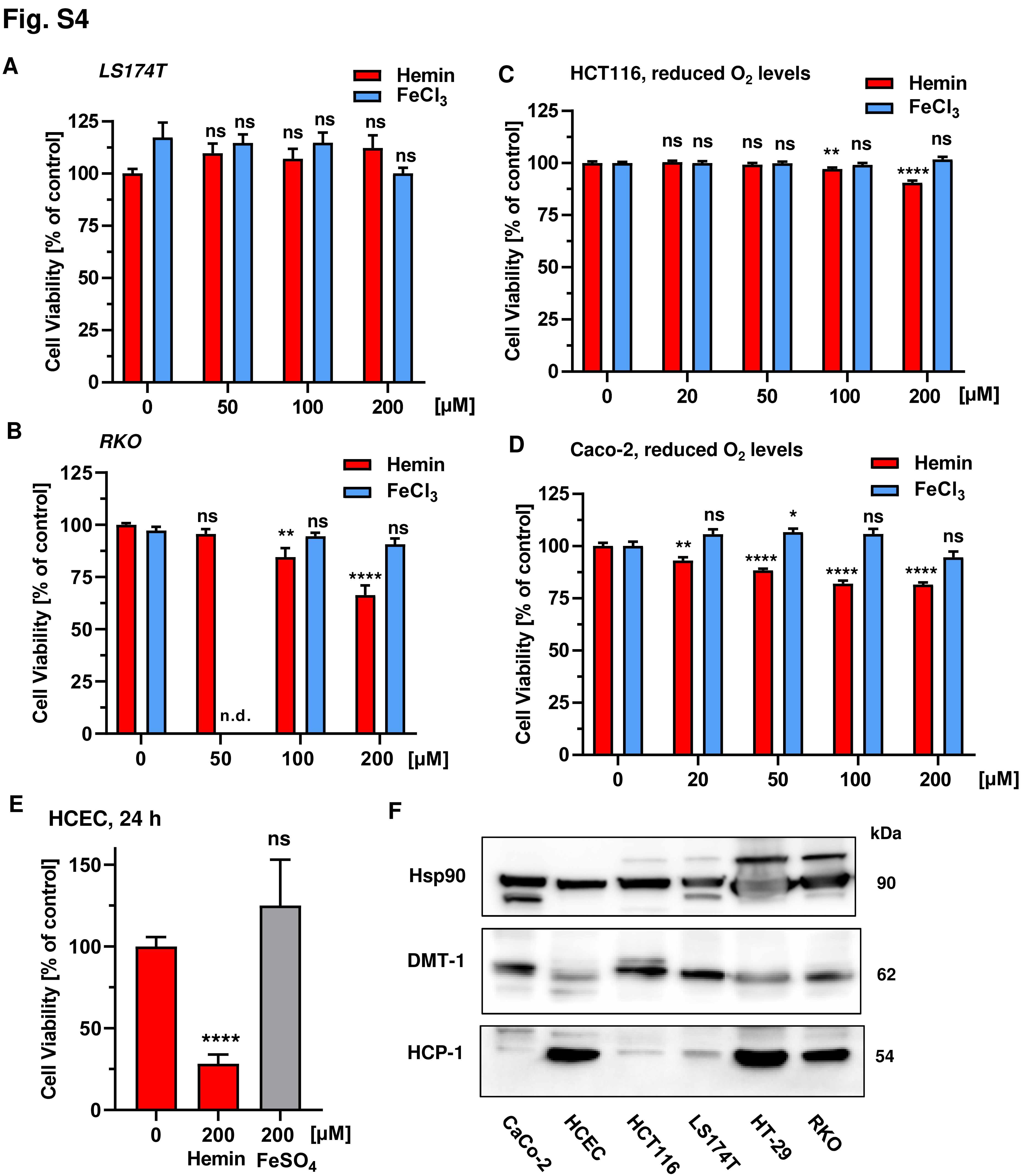

Supplement: Supplementary file 5 — Figure S4 [file 41419_2020_2950_MOESM5_ESM.tif]

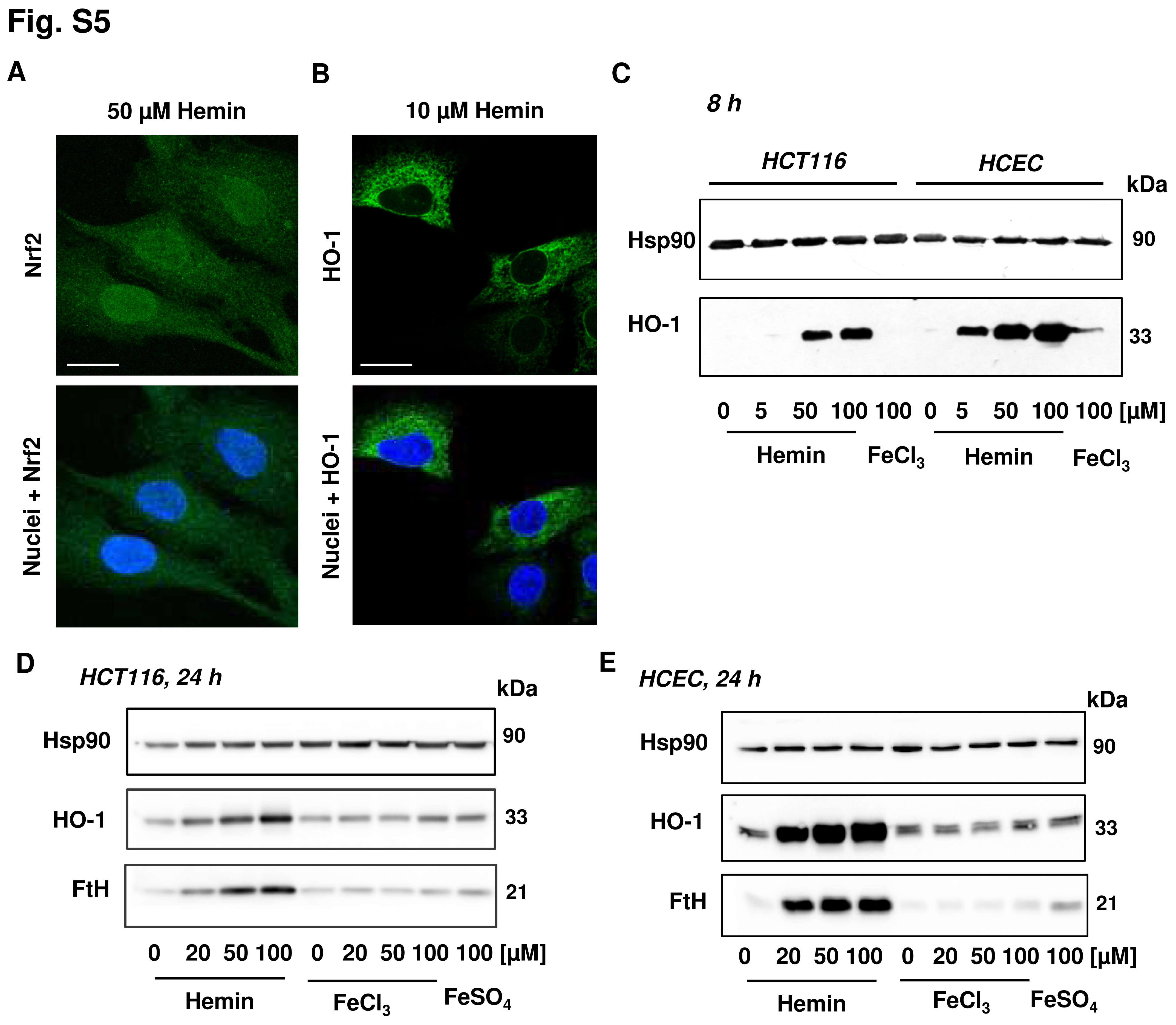

Supplement: Supplementary file 6 — Figure S5 [file 41419_2020_2950_MOESM6_ESM.tif]

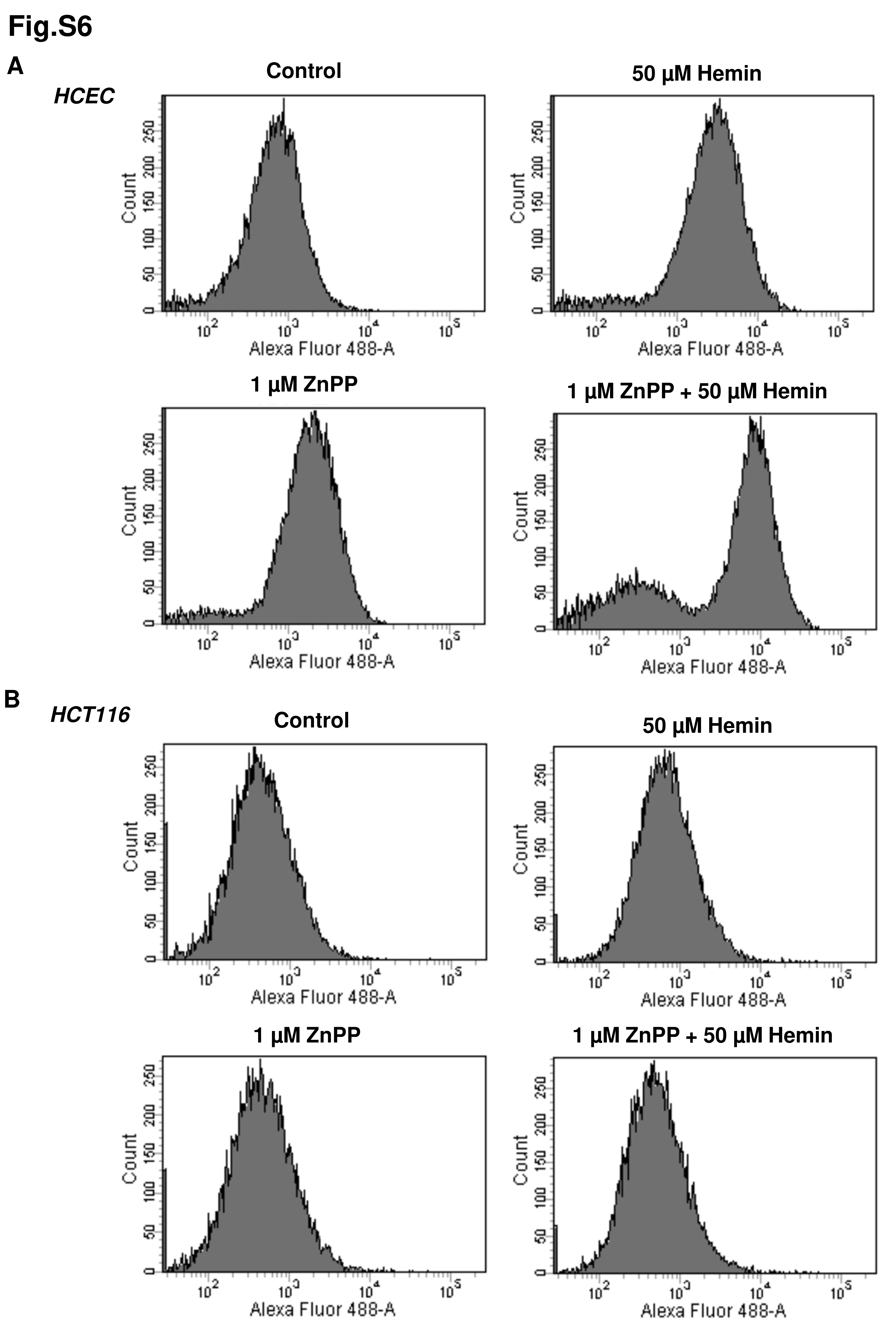

Supplement: Supplementary file 7 — Figure S6 [file 41419_2020_2950_MOESM7_ESM.tif]

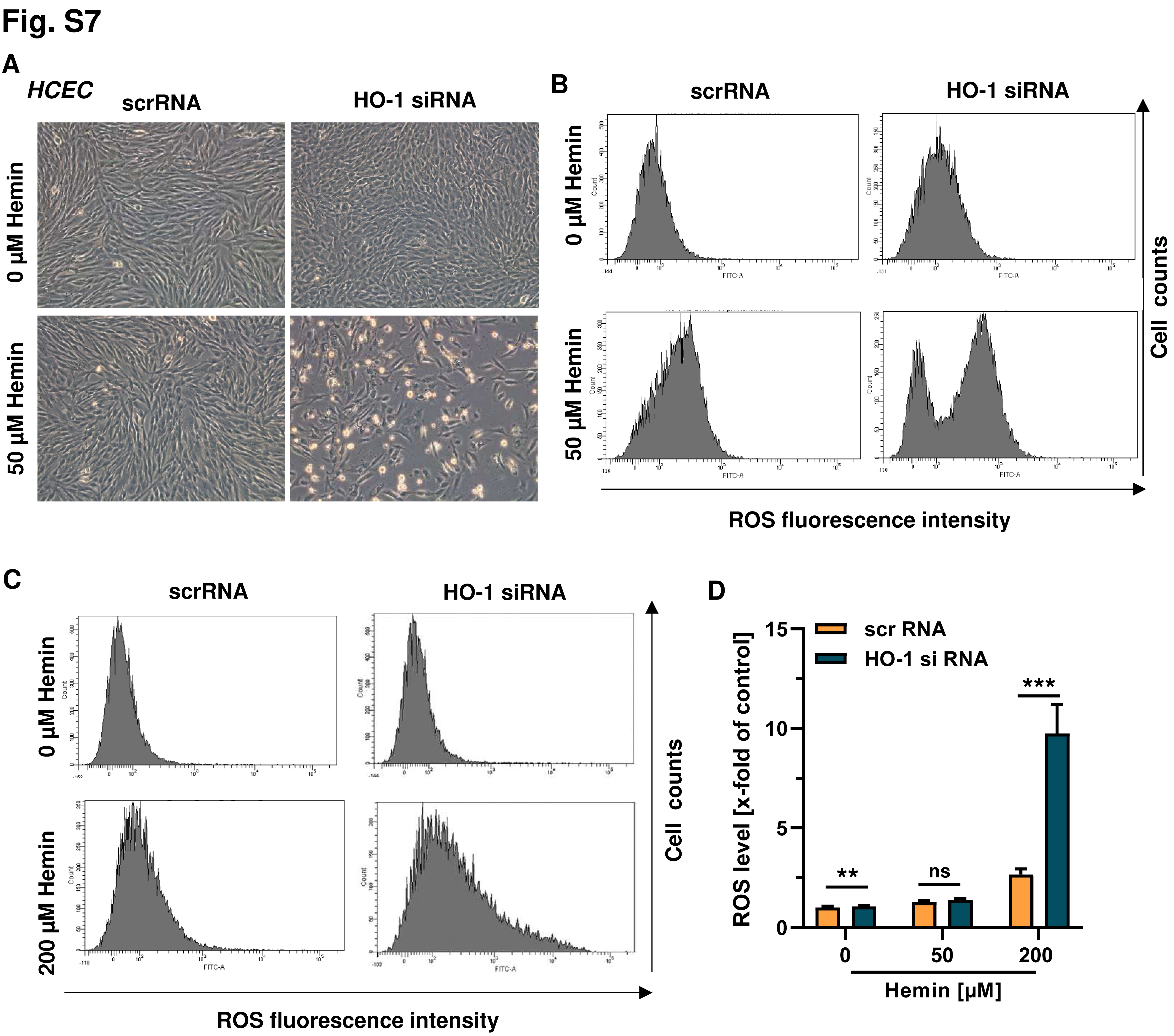

Supplement: Supplementary file 8 — Figure S7 [file 41419_2020_2950_MOESM8_ESM.tif]

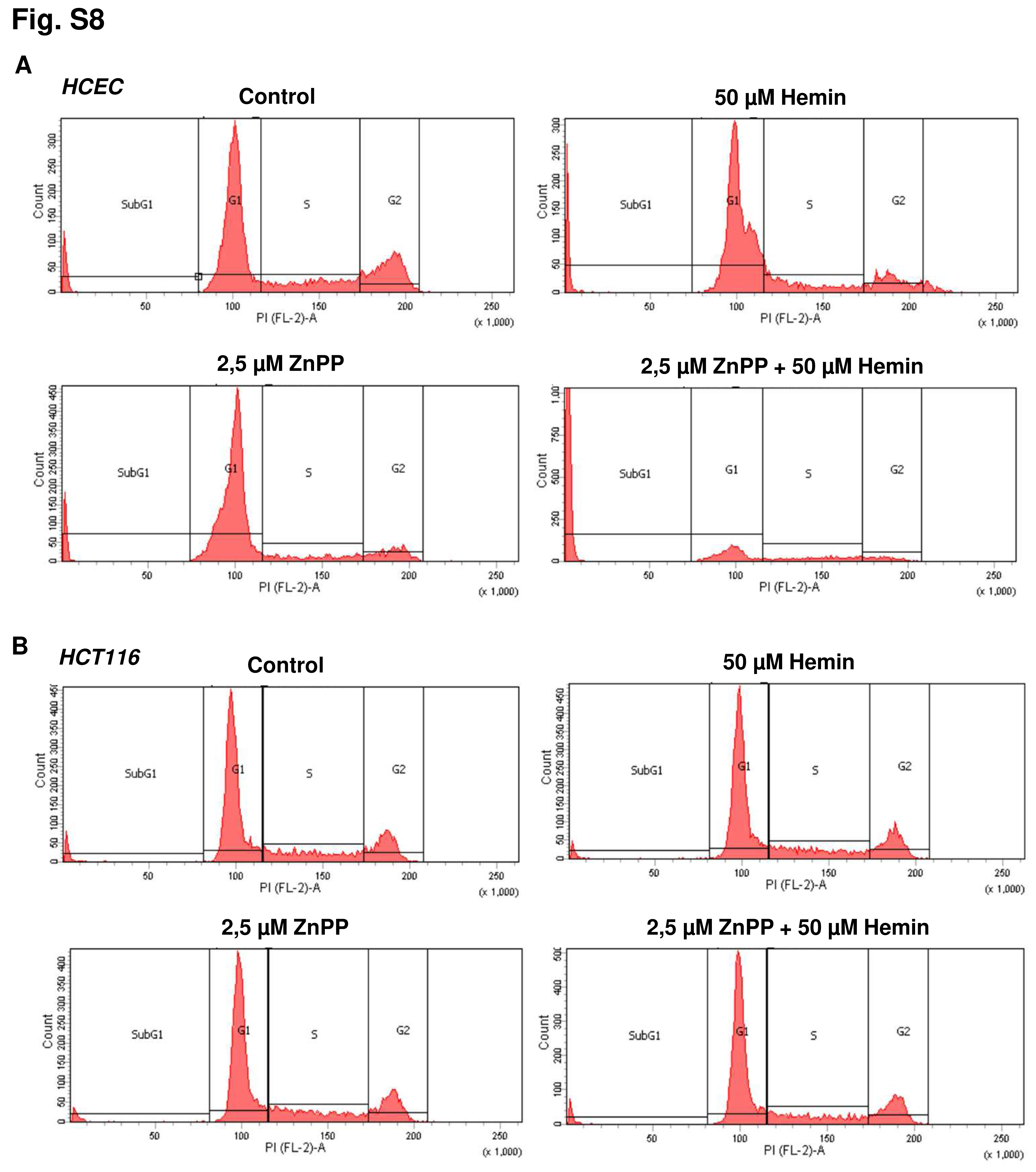

Supplement: Supplementary file 9 — Figure S8 [file 41419_2020_2950_MOESM9_ESM.tif]

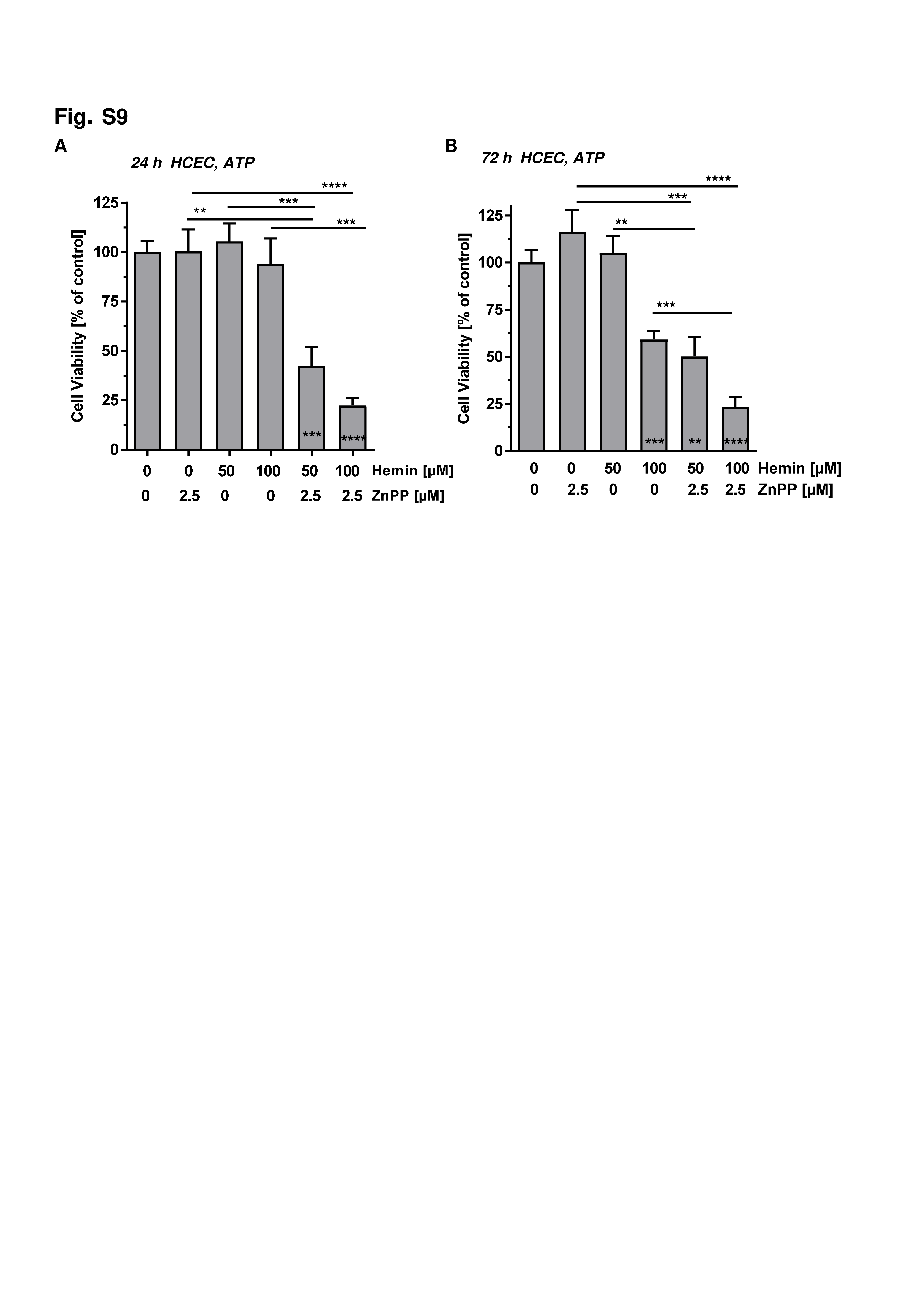

Supplement: Supplementary file 10 — Figure S9 [file 41419_2020_2950_MOESM10_ESM.tif]
